# Hybrid photon–phonon blockade

**DOI:** 10.1038/s41598-022-21267-4

**Published:** 2022-10-21

**Authors:** Shilan Abo, Grzegorz Chimczak, Anna Kowalewska-Kudłaszyk, Jan Peřina, Ravindra Chhajlany, Adam Miranowicz

**Affiliations:** 1 Institute of Spintronics and Quantum Information, Faculty of Physics, Adam Mickiewicz University, 61-614 Poznan, Poland; 2 Joint Laboratory of Optics of Palacký University and Institute of Physics of CAS, Faculty of Science, Palacký University, 17. listopadu 12, 771 46 Olomouc, Czech Republic

**Keywords:** Single photons and quantum effects, Quantum mechanics

## Abstract

We describe a novel type of blockade in a hybrid mode generated by linear coupling of photonic and phononic modes. We refer to this effect as hybrid photon–phonon blockade and show how it can be generated and detected in a driven nonlinear optomechanical superconducting system. Thus, we study boson-number correlations in the photon, phonon, and hybrid modes in linearly coupled microwave and mechanical resonators with a superconducting qubit inserted in one of them. We find such system parameters for which we observe eight types of different combinations of either blockade or tunnelling effects (defined via the sub- and super-Poissonian statistics, respectively) for photons, phonons, and hybrid bosons. In particular, we find that the hybrid photon–phonon blockade can be generated by mixing the photonic and phononic modes which do not exhibit blockade.

## Introduction

Photon blockade (PB)^1^, also referred to as optical state truncation (see reviews in Refs.^2,3^), or nonlinear quantum scissors (for a review see Ref.^4^) is an optical analogue of Coulomb’s blockade. Specifically, it refers to the effect in which a single photon, generated in a driven nonlinear system, can block the generation of more photons. The light generated by an ideal (or ‘true’) PB exhibits both sub-Poissonian photon-number statistics and photon antibunching. But even if one of these properties is satisfied, the term PB is often used.

PB has been demonstrated experimentally in various driven nonlinear systems with single^5–11^ and two^12,13^ resonators, in a bimodal cavity^14^, or even in cavity-free systems^15^. Experimental platforms where PB was observed include: cavity quantum electrodynamics (QED) with Fabry-Perot cavities^5^, photonic crystals^6^, and whispering-gallery-mode cavities^16^, as well as circuit QED^7,8^. Note that the possibility of producing a single-photon state in a driven cavity with a nonlinear Kerr medium was predicted already in Refs.^17–19^, but only the publication of Ref.^1^, where the term ‘photon blockade’ was coined, has triggered much interest in studying this effect both theoretically and experimentally. Arguably, many studies reported already in the 1970s and 1980s on photon antibunching and sub-Poissonian light (see, e.g., reviews in Refs.^20–22^ and references therein) are actually about PB-related effects, although such a relation (to the optical analogue of Coulomb’s blockade) was not mentioned explicitly there.

In addition to the original idea of using PB as a single-photon turnstile device with single^1,16,23^ or multiple^24^ outputs, PB can have much wider applications in quantum nonlinear optics at the single-photon level, including single-photon induced nonlinear effects, quantum noise reduction via antibunching of photons, simulations of nonreciprocal nonlinear processes, or studying chirality at exceptional points for quantum metrology, etc.

A number of generalisations of the standard single-PB effect were proposed, which include: (1) two- and multi-photon versions of PB, as first predicted in Refs.^25,26^ and demonstrated experimentally in Refs.^11,27^; (2) unconventional PB as predicted in Ref.^28^ and experimentally demonstrated in Refs.^12,13^; (3) conventional and unconventional nonreciprocal PB effects as predicted in Refs.^29,30^ and (at least partially) confirmed experimentally in Ref.^31^; (4) state-dependent PB^32^, (5) exceptional PB^33^, and (6) linear quantum scissors based on conditional measurements for: single-PB^34–36^, which was experimentally demonstrated in Ref.^37^, as well as two-PB^38^, and multi-PB^39,40^ using multiport Mach–Zehnder interferometers^41^. This probabilistic approach to PB enables also nondeterministic quantum teleportation and more selective optical-state truncations, e.g, hole burning in the Hilbert space^42^. Concerning example (2), note that PB in two driven Kerr resonators was first studied in Refs.^43,44^, but only for relatively strong Kerr nonlinearities. Surprisingly, PB remains in such two-resonator systems even for extremely weak Kerr nonlinearities, as first predicted in Ref.^28^ and explained via destructive quantum interference in Ref.^45^. This effect is now referred to as unconventional PB^46^.

Here we study phonon blockade^47^, which is a mechanical analogue of the mentioned blockade effects, i.e., the blockade of quantum vibrational excitations of a mechanical resonator. This effect has not been demonstrated experimentally yet. However, a number of experimentally feasible methods have been proposed for measuring it, including a magnetomotive technique^47^, an indirect measurement of phonon correlations via optical interferometry^48^, or by coupling a mechanical resonator to a qubit, which is used not only for inducing the resonator nonlinearity, but also to detect the blockade effect itself, i.e., by measuring qubit’s states^49^. Among possible applications of phonon blockade, we mention: testing nonclassicality of meso- or macroscopic mechanical systems^47^ and studying single-phonon optomechanics, in addition to offering a source of single- or multiple phonons^50,51^.

PB can be changed into light transmission^52^, e.g., by photon-induced tunnelling (PIT)^6^. This is another nonclassical photon-number correlation phenomenon, in which the probability of observing more photons in a higher manifold of the system increases with the generation of the first photon near the resonance frequency of the system. Multi-PIT effects were also predicted^29^, including those generated by squeezing^53^.

For simplicity, we use here the abbreviation PB, when referring to the blockade of not only photons, but also of phonons or hybrid photon–phonon bosons. The precise meaning can be found from its context, e.g., when we refer to a specific mode, including the optical (*a*), mechanical (*b*), or hybrid (*c*) modes. Analogously, PIT denotes a given particle-induced tunnelling among the three types of excitations.

Nanomechanical resonators can coherently interact with electromagnetic radiation^54^, and quantum correlations between single photons and single phonons were studied for a single entangled photon–phonon pair^55^ or via photon and phonon blockade effects in optomechanical systems^56^. A mechanical switch between PB and PIT has been studied recently^57^. PB and PIT effects in systems comprising mechanical and optical resonators, which are characterised by the same or similar bare frequencies, to our knowledge, have not been studied experimentally yet, although they seem to be experimentally feasible and, thus, they are at focus of this paper.

Crucial signatures of PB and PIT can be observed by measuring the second-order correlation function, $g^{(2)}(0)$. Specifically for photons, (1) the condition of $g^{(2)}(0)<1$ defines the sub-Poissonian photon-number statistics (also referred to as zero-delay-time photon antibunching), which indicates the possibility of observing PB, while (2) the condition $g^{(2)}(0)>1$, defines the super-Poissonian statistics (also referred to as zero-delay-time photon bunching), which is a signature of PIT in a given system. To observe the ‘true’ effects of PB and PIT, also other criteria should be satisfied, such as nonzero-delay-time photon antibunching and higher-order sub-Poissonian photon-number statistics. Indeed, an ideal conventional PB, which can be served as a single-photon source, usually should also be verified by studying higher-order correlation functions, $g^{(n)}(0)$ for $n>2$. For example, in case of single-PB (1PB) conditions $g^{(2)}(0)<1$ and $g^{(n)}(0)<1$ for $n>2$ should be fulfilled.

PB can be verified also in other ways via demonstrating, e.g., a staircase-like dependence of the mean photon number (or measured power transmitted through a nonlinear resonator) on the energy spectrum of the photons incident on the resonator^8,52^. Such a dependence is the photon analogue of the Coulomb staircase. All of the above criteria are just necessary but not sufficient conditions for demonstrating PB. A sufficient condition could be, e.g., showing a high fidelity of a given generated light (with a nonzero mean photon number) to an ideally truncated two-dimensional state, which is the closest to the generated one. This approach was applied in, e.g.,^26,35,36^. The latter two types of PB tests are, however, are not applied in this paper.

Conventional single-PB prevents the absorption of a second photon with a specific frequency due to the nonlinearity of a given system. Such a nonlinearity can be described by a Kerr-type interaction and/or can induced by an atom (real or artificial) coupled to a resonator. An artificial atom can be realised by, e.g., a quantum dot^23,58,59^ in cavity QED^10^ or a superconducting qubit or qudit in circuit QED^52^.

Unconventional PB, which is induced by destructive interference, operates better for very low (or even extremely low) mean photon numbers^12,13^. This can be disadvantageous by considerably decreasing the probability of generating a single photon. But, at the same time, it can be an advantage, because a very small mean photon number usually reduces the chance of generating multi-photon states and inducing higher-order coherence. This is not always the case, and even if the probability of observing two photons is suppressed, higher-order coherence might be enhanced, leading to the generation of multi-photon states^46^.

In this paper, we consider an optomechanical system, which generates photonic and phononic modes. Then we apply a balanced linear coupling transformation to the these modes to create hybrid modes (also referred to as supermodes). We study the interplay between photons and phonons resulting in their nonclassical number correlation effects. Thus, we find such system parameters to observe either PB or PIT in the four modes. In particular, we predict PB in one of the hybrid modes, but not in the individual (photon and phonon) modes, i.e., this PB is created from the two modes, which do not exhibit PB. We refer to this effect as hybrid photon–phonon blockade, which is the main result reported here.

Specifically, we define hybrid photon–phonon blockade as the blockade of hybrid-mode bosons (polaritons) obtained by coupling photons of an optical or microwave mode with phonons of a mechanical mode by a balanced linear coupler. The idea and criteria for testing this type of blockade are analogous to those for other known blockade effects (e.g., of photons, phonons, or magnons), but it is predicted for another type of bosons. We show that this hybrid blockade can occur by coupling the modes, which exhibit neither photon blockade nor phonon blockade.

To show this effect we analyse the system of two linearly-coupled resonators: a superconducting microwave resonator (SMR), which might be a transmission line resonator, and a micromechanical resonator, referred to as a quantum drum (QD), which is capacitively coupled to the SMR. To generate any kind of PB (including unconventional PB), one needs to incorporate a nonlinearity into a given system^17,60,61^. This can be done by coupling one of the resonators (e.g., the SMR) to a qubit (e.g., an artificial superconducting two-level atom). We also assume that the system is driven either at the QD or the SMR as described in detail in the next section.

The paper is organised as follows: first, the hybrid optomechanical system and its Hamiltonians are introduced. We also define the hybrid photon–phonon modes, which can be generated by the balanced linear coupling of photonic and phononic modes. Then, we study the correlation effects in the photonic, phononic, and one of the hybrid modes in the system driven at either the optical or mechanical resonator, respectively, for experimentally feasible parameters specified in “Methods”. We then predict and analytically explain the generation of unconventional hybrid-mode blockade via a non-Hermitian Hamiltonian method. We systematically study different weaker and stronger criteria for observing blockade and tunnelling effects in our system. We also find all the eight combinations of the conventional blockade and tunnelling effects in the three modes. In particular, we find a surprising effect that the hybrid-mode photon–phonon blockade can be generated by mixing the photonic and phononic modes exhibiting tunnelling effects. In addition to this study of the second-order correlation effects, we discuss also higher-order effects and their classification in “Methods”. Moreover, we discuss two types of schemes for measuring photon–phonon correlations in hybrid modes. Finally, we summarise our results and indicate their potential applications.

## The system and Hamiltonians

**Figure 1:**
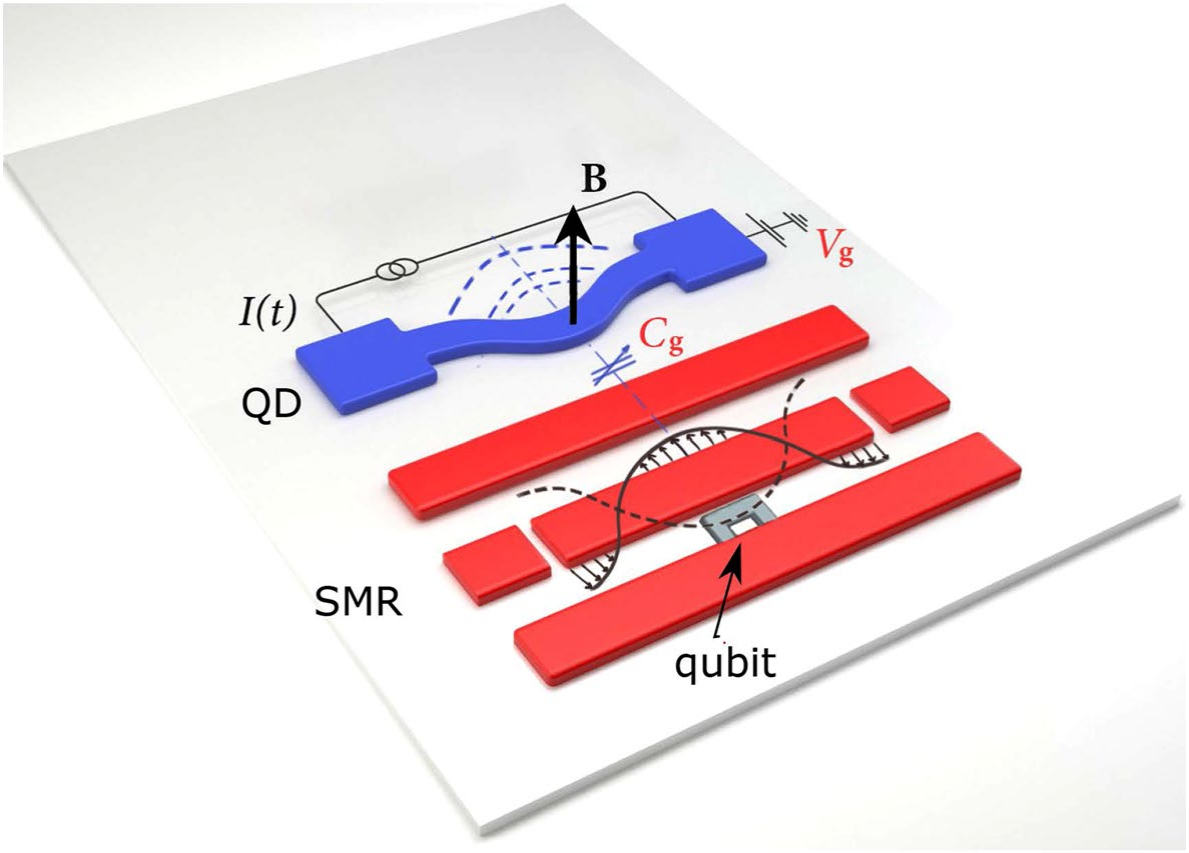
Schematics of the discussed circuit-QED-based realisation of the considered hybrid optomechanical system. It consists of a superconducting qubit embedded in a superconducting microwave resonator (SMR), e.g., a transmission-line resonator, to induce its nonlinearity. A quantum micromechanical resonator, which is referred to as a quantum drum (QD), is coupled to the SMR with a tunable capacitor $C_g$. We assume that the system is driven either at the SMR or QD. Dashed semicircular curves visualise that the QD is oscillating. The driving and motion detection of the QD can be realised by controlling the static magnetic field *B*, potential $V_g$, and alternating current *I*(*t*), as described in Ref.^47^ for detecting phonon blockade.

Figure 1 shows the schematics of the studied hybrid system, which consists of a superconducting two-level artificial atom (a qubit) embedded in a waveguide and coupled to an SMR, which might be a transmission-line resonator. This qubit induces anharmonicity in the SMR, which is crucial for observing PB. Our setup includes also a microwave-frequency mechanical resonator (a QD), which is capacitively coupled to the SMR. The nonlinearity of the QD is induced indirectly by the linear coupling of the QD to the effectively nonlinear SMR.

The free Hamiltonian of the SMR is $H_a=\hbar \omega _{_\mathrm{SMR}} a^\dagger a$, where $\omega _{_\mathrm{SMR}}$ is its resonance frequency (assumed here of the order of tens of GHz) and $a\,(a^{\dagger })$ is the photon annihilation (creation) operator. We can reasonably assume the SMR quality factor as $Q_{_\mathrm{SMR}}\approx 10^4$. The free Hamiltonian of the QD is $H_b=\hbar \omega _m b^\dagger b$, where $\omega _m$ is its resonance frequency and $b\,(b^{\dagger })\,$ is the phonon annihilation (creation) operator. In our numerical simulations, we set $\omega _m/2\pi =7.8$ GHz and the QD quality factor as $Q_m\approx 260$. Moreover a two-level quantum system has the ground state $| g \rangle$ and the excited state $| e \rangle$ with transition frequency $\omega _q$ (set here of the order of $\omega _m$ and $\omega _{_\mathrm{SMR}}$). The free qubit Hamiltonian is described as $H_q= \hbar \omega _{q} \sigma _{+} \sigma _{-}$, where $\sigma _{+}=| e \rangle \langle g |$ ($\sigma _{-}=| g \rangle \langle e |$) is the atomic raising (lowering) operator. Thus, the total free Hamiltonian of the system is $H_0=H_a+H_b+H_q$. The complete Hamiltonian (without driving) of our coupled system can be given by ($\hbar =1$)

1 $$\begin{aligned} H'_{\pm }= H_0+g(a^\dagger \sigma _{-} +a\sigma _{+}) +g_r(b+b^\dagger )a^\dagger a+g_l(a \pm a^{\dagger })(b+b^{\dagger }), \end{aligned}$$

which includes the three coupling terms: (1) the Jaynes–Cummings term describing the interaction between the SMR and qubit under the rotating-wave approximation (RWA); (2) the radiation-pressure term with coupling strength $g_r$; and (3) the Hopfield-type nonlinear coupling term with strength $g_l$. The $g_l$ coupling can be realized via a capacitor, as shown in Fig. 1 and explained in a more detail in Ref.^48^ for a similar system. Note that the $g_l$ term describes canonical position–position (momentum–position) interactions, where $g_l$ is real (imaginary) for $H'_{+}$ ($H'_{-}$). These interactions can be interchanged by adding the $\pi /2$ phase to ${a,a^\dagger }$, i.e., $a\rightarrow i a$ and $a^\dagger \rightarrow -i a^\dagger$. This extra phase does not change number correlations in the modes *a* and *b*. In typical ranges of parameters of analogous superconducting circuits^62^, the $g_l$ term is dominant, so the radiation-pressure term can be neglected^63^. Moreover, although the counter-rotating terms $ab\pm b^\dagger a^\dagger$, which appear in the $g_l$-interaction, play an important role in the ultrastrong and deep-strong coupling regimes^64^, but they can be safely omitted under the RWA, which is valid in the weak and strong coupling regimes. Indeed, the latter regimes are solely studied in this paper, as discussed below. Then the Hopfield nonlinear $g_l$-interaction becomes effectively linearised. Thus, Hamiltonian (1) reduces to

2 $$\begin{aligned} H_{\pm }= & {} H_0 +g(a^\dagger \sigma _{-} +a\sigma _{+}) +f(a b^{\dagger } \pm a^{\dagger }b), \end{aligned}$$

where the linear-coupling strength is denoted by *f*, which replaces the symbol $g_l$. Analogously to $g_l$, *f* is real (imaginary) for $H_{+}$ ($H_{-}$). In the following, for simplicity, we focus on studying the canonical position–position interactions between the modes *a* and *b*, as described by $H_{+}$. The eigenstates of Hamiltonian $H_{\pm }$ can be referred to as atomic-optomechanical polaritons or atom-cavity-mechanics polaritons^65^. It is clear that Hamiltonians $H_{\pm }$ conserve the polariton number,

3 $$\begin{aligned} N_\mathrm{polariton}=a^\dagger a + b^\dagger b + \sigma _+\sigma _-, \end{aligned}$$

which is the total number of excitations. Thus, $H_{\pm }$ can be diagonalised in each subspace (or manifold) $\mathcal{H}^{(n)}$ with exactly *n* polaritons.

The RWA is fully justified assuming both (1) the weak- or strong-couplings and (2) small detunings between the SMR and QD, and the SMR and qubit (see, e.g., Ref.^66^). We stress that these conditions are fully satisfied for the parameters applied in all our numerical calculations in this paper. Thus, the Jaynes–Cummings and frequency-converter (or linear-coupler) models can be applied. However, the RWA cannot be applied in the ultrastrong and deep-strong coupling regimes, as defined by $g> 0.1\,\omega _{i}$ and $g>\omega _{i}$, respectively^64^, where $i=\mathrm{SMR}, m, q$. In these regimes, the quantum Rabi and Hopfield models cannot be reduced to the Jaynes–Cummings and frequency-converter models, respectively. However, we study the system for the parameters specified in Eqs. (28)–(30), for which the ratios of the coupling strengths and frequencies, $f/\omega _i$ and $g/\omega _i$, are $<0.002$. So, the system is in the strong-coupling regime, and far away from the border line with the USC regime. Moreover, the chosen detunings are $|\omega _{_\mathrm{SMR}}-\omega _m|/\omega _{_\mathrm{SMR}} \le 2.6 \times 10^{-3}$ and $|\omega _{_\mathrm{SMR}}-\omega _q|/\omega _{_\mathrm{SMR}} < 8 \times 10^{-4}$. Thus, it is clearly seen that we can safely apply the RWA. Anyway, as a double test, we have calculated time-dependent second-order correlation functions for the Hamiltonian $H'_{\pm }$ and $H_{\pm }$ for the parameters set in Eqs. (28)–(30) for various evolution times assuming classical drives (as specified below) and no dissipation. And we have found that the differences between the correlation functions calculated for the models with and without the RWA are negligible on the scale of figures. The inclusion of dissipation in the system makes such differences even smaller.

We assume that an optical pump field of frequency $\omega _{p}$ is applied either to the SMR mode *a*, as described by

4 $$\begin{aligned} H^{(a)}_\mathrm{drv}(t)=\eta _{a}(e^{i\omega _p t}a+e^{-i\omega _{p}t}a^{\dagger }), \end{aligned}$$

or to the QD mode *b*, as given by

5 $$\begin{aligned} H^{(b)}_\mathrm{drv}(t)=\eta _{b}(e^{i\omega _p t}b+e^{-i\omega _{p}t}b^{\dagger }), \end{aligned}$$

to drive (excite) the system (with coupling strength $\eta _{a}$ or $\eta _{b}$) from its ground state and to induce the emission of photons and phonons. Thus, the total Hamiltonian becomes

6 $$\begin{aligned} H^{(n)}(t)=H_+ +H^{(n)}_\mathrm{drv}(t) \qquad (n=a,b). \end{aligned}$$

Direct driving of the QD can be implemented by a weak-oscillating current, as considered in Refs.^47,48^, where the drive strength $\eta _b$ is proportional to the current amplitude *I*(*t*) and the magnetic field *B* shown in Fig. 1. The SMR can be driven in circuit-QED systems in various ways^62^.

Note that by driving directly the SMR (or alternatively the QD), one also indirectly drives the QD (SMR) through the capacitive coupling $C_g$, as shown in the scheme in Fig. 1. So, by referring to the SMR- or QD-driven systems, we indicate only the resonator, which is directly pumped, although finally both resonators are driven.

The inclusion of an additional nonlinearity in the QD and/or applying drives to the qubit(s) and both resonators is not essential for the prediction of hybrid blockade, but this could enable achieving stronger photon–phonon antibunching and more sub-Poissonian statistics.

Considering the case, where the pump field drives only the SMR, to remove the time dependence of the Hamiltonian $H^{(n)}(t)$ and to obtain its steady-state solution, we transform the system Hamiltonian into a reference frame rotating at frequency $\omega _p$.

We apply the unitary transformation $U_R(t)=\exp \left( -i N_\mathrm{polariton}\omega _p t \right)$ to $H^{(n)}$ according to the general formula

7 $$\begin{aligned} H^{(n)}_\mathrm{rot}= U_R^\dagger H^{(n)} U_R -i U_R^\dagger \frac{\partial }{\partial t} U_R. \end{aligned}$$

Thus, $H^{(a)}(t)$ reduces the time-independent SMR-driven Hamiltonian:

8 $$\begin{aligned} H' &\equiv {} H^{(a)}_\mathrm{rot} = \Delta _{_\mathrm{SMR}}a^\dagger a+\Delta _{m} b^\dagger b +\Delta _{q} \sigma _{+} \sigma _{-} \nonumber \\&\quad +g(a^\dagger \sigma _{-} +a\sigma _{+})+f(a^{\dagger }b+a b^{\dagger })+\eta _{a}(a+a^{\dagger }), \end{aligned}$$

where $\Delta _{i}=\omega _{i}-\omega _{p}$ for $i=a,b,q$. So, in particular, $\Delta _{b}\equiv \Delta _{m}$ ($\Delta _{a}\equiv \Delta _{_\mathrm{SMR}}$) is the mechanical (microwave) resonator frequency detuning with respect to the pump frequency. Analogously, in the same rotating frame, $H^{(b)}(t)$ reduces to the QD-driven Hamiltonian:

9 $$\begin{aligned} H' &'\equiv {} H^{(b)}_\mathrm{rot} = \Delta _{_\mathrm{SMR}}a^\dagger a+\Delta _{m} b^\dagger b +\Delta _{q} \sigma _{+} \sigma _{-} \nonumber \\&\quad +g(a^\dagger \sigma _{-} +a\sigma _{+})+f(a^{\dagger }b+a b^{\dagger })+\eta _{b}(b+b^{\dagger }). \end{aligned}$$

We recall that Eqs. (8) and (9) are directly derived from Eq. (6) for $H_+$ given in Eq. (2). Moreover, $H_+$ is derived from Eq. (1) assuming the RWA, which is justified for small detunings in the weak- and strong-coupling regimes, which are the only numerically studied regimes in this paper, as emphasised above. Indeed, the studied ranges of parameters guarantee the system evolution is far from the USC regime. Note that the Hamiltonian $H'_+$ in (1) for $g_r=0$ with an additional drive term $H^{(n)}_\mathrm{drv}(t)$ can be transformed, according to Eq. (7), to $H^{(n)}_\mathrm{rot}$ given in Eqs. (8) and (9) but with the additional term $f[ a b \exp (2i\omega _p t)+\mathrm{h.c.}]$. In all our numerical calculations we set $\omega _p$ of the order of GHz. Thus, the effect of this rapidly oscillating term is negligible compared to all the other terms in the Hamiltonians. Moreover, we have also assumed that the optomechanical term $g_r$ is negligible. In general, this assumption is not necessary, because the $g_r$ term can be reduced (in the red-detuned regime) to an interaction term describing a linear coupler (or a beam splitter), which can be combined with the *f* term. Anyway, for simplicity concerning both theory and potential experiments, we set $g_r=0$. We have also assumed that the system is driven at either the mechanical or optical mode to obtain effectively time-independent Hamiltonians in a rotating frame. This simplification would not be directly possible by considering the system driven simultaneously at both modes with different frequencies.

**Figure 2:**
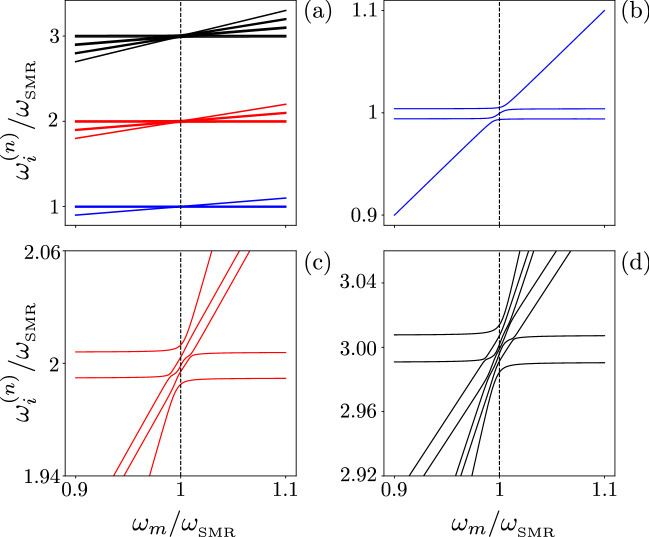
Energy levels $\omega _n$ versus the QD frequency $\omega _m$ in units of the SMR frequency $\omega _{_\mathrm{SMR}}$ for the Hamiltonian Eq. (2) with the parameters given in Eq. (28) and $g=7.5\gamma$. The three manifolds of the lowest energy levels in panel (**a**) are zoomed in panels (**b**–**d**) near the resonance $\omega _m=\omega _{_\mathrm{SMR}}$ to reveal the anti-crossing of energy levels. Here, $\omega ^{(n)}_i$ (with $n=1,2,3$) denotes the frequencies of the $n\hbox {th}$ manifold.

Figure 2 shows the structure of the energy spectrum for the hybrid system Hamiltonian (2). To study the sub-Poissonian light generation in hybrid modes, we apply to the SMR and QD modes a balanced linear coupling transformation, which is formally equivalent to a balanced (50/50) beam splitter (BS). This transformation creates the hybrid (or cross) photon–phonon modes:

10 $$\begin{aligned} c=\frac{a+b}{\sqrt{2}},\quad d=\frac{a-b}{\sqrt{2}}, \end{aligned}$$

for the system described by $H_+$ and related Hamiltonians. Note that if this BS transformation is modified as $a\rightarrow -i a$ and $a^\dagger \rightarrow i a^\dagger$ [which compensate the extra $\pi /2$ phase introduced below Eq. (1)] then all our predictions of number correlations shown in various figures for the hybrid mode *c* (in addition to those for the modes *a*, *b*, and *d*) are the same as those for the model described by $H_-$.

Thus, the Hamiltonian $H'$ after the BS transformation reads

11 $$\begin{aligned} H'_\mathrm{BS}= & {} \Delta _c c^\dagger c+\Delta _d d^\dagger d + \Delta _q \sigma _{+}\sigma _{-} +\delta (c^\dagger d +d^\dagger c) \nonumber \\&+\frac{1}{\sqrt{2}}\left[ \eta _{a}(c+c^\dagger ) +\eta _{a}(d+d^\dagger ) + g(c^\dagger \sigma _{-}+c\sigma _{+}) + g(d^\dagger \sigma _{-} +d \sigma _{+})\right] , \end{aligned}$$

which describes the qubit interacting with two hybrid modes *c* and *d*, where $\Delta _{c,d}=(\omega _{_\mathrm{SMR}}+ \omega _m)/2-\omega _p\pm f$ and $\delta =(\omega _{_\mathrm{SMR}}- \omega _m)/2$. It is seen that the two modes *c* and *d* have no direct coupling if $\omega _m=\omega _{_\mathrm{SMR}}$.

The dynamics of an open system in the presence of losses under the Markov approximation can be described within the Lindblad approach for a system reduced density matrix $\rho$ satisfying the standard master equation,

12 $$\begin{aligned} \frac{\partial \rho }{\partial t}= -i[H, \rho ]+\kappa _a \mathscr {D}[a]\rho +\kappa _b \mathscr {D}[b]\rho +\gamma \mathscr {D}[\sigma ]\rho , \end{aligned}$$

which is given in terms of the Lindblad superoperator $\mathscr {D}[O]\rho = \frac{1}{2}(2O\rho O^\dagger -\rho O^\dagger O-O^\dagger O\rho )$, where $\kappa _a$, $\kappa _b,$ and $\gamma$ are the decay rates for the SMR, QD, and qubit, respectively.

All our numerical calculations and their analyses are given for the system parameters, which satisfy the conditions for the weak or strong-coupling regimes and for small detunings between the SMR, QD, and qubit. Thus, we can safely apply the standard master equation given in Eq. (12). Of course, if one considers Eq. (1) for the system in the USC or deep-coupling regimes, then the master equation in Eq. (12), should be replaced by a generalised one, e.g., of Refs.^64,67–69^.

We also note that the application even of a single classical drive to the Jaynes–Cummings model in the strong-coupling regime effectively creates counter-rotating terms, which can induce a variety of USC effects, as shown explicitly in Ref.^70^. Thus, to confirm the validity of our results, we have applied the generalised formalism described in Ref.^67^, which is valid for arbitrary light-matter coupling regimes, including the weak-, strong-, and USC regimes. In particular, we calculated the correlation functions $g^{(n)}(0)$ defined in terms of the positive- ($X^+_n$) and negative- [$X^-_n=(X^+_n)^{\dagger }$] frequency components of the canonical position operators: $X_a = a + a^{\dagger }$ for photons, $X_b=b+b^{\dagger }$ for phonons, and $X_c=c+c^{\dagger }$ for hybrid-mode bosons in the qubit-SMR-QD dressed basis. We calculated the steady states of the system by solving numerically the generalised master equation of Ref.^67^ for the Hamiltonians $H'$ and $H''$. As expected from general considerations, our numerical calculations for the parameters set in Eqs. (28)–(30) using the standard and generalised formalisms based on $H'$ (as well as $H''$) give effectively the same results.

In our simulations, we assume that the system is prepared in the ground state $| n=0, g \rangle | m=0 \rangle$ (i.e., with no photons in the SMR, no phonons in the QD, and the qubit is in the ground state), such that a given pump laser can drive the SMR photons in the microwave frequency range. Note that the choice of initial states affects the short-time evolution of our system, but has no effect on the steady-state solutions in the time limit, assuming the single-photon and single-phonon damping channels, as described in Eq. (12). However, as shown in Ref.^32^, initial states of a system can indeed affect steady states of the system, thus can also change PB, in case of quantum engineered dissipation channels allowing for, e.g., two-photon dissipation only.

In the following sections, we show that it is possible to observe both PB and PIT in the hybrid mode in the weak, mediate, and strong coupling regimes compared to the decay rates of the SMR, QD, and qubit. In particular, we show that the system can generate the hybrid photon–phonon modes with strongly sub-Poissonian (or super-Poissonian) statistics by mixing the SMR and QD modes with strongly super-Poissonian (or sub-Poissonian) statistics.

## Hybrid-mode blockade in the SMR-driven system

Here we analyse in detail various blockade and PIT effects in the SMR-driven dissipative system described by the Hamiltonian $H'$ and the master equation (12) for the parameters specified in Eq. (28).

Photon/phonon-number statistics of the modes generated by our hybrid system can be described quantitatively by calculating the zero-delay-time $k\hbox {th}$-order correlation function ($k\hbox {th}$-order intensity autocorrelation function),

13 $$\begin{aligned} g_z^{(k)}(0)=\lim _{t\rightarrow \infty }\frac{\langle z^{\dagger k}(t) z^k(t) \rangle }{\langle z^\dagger (t) z(t)\rangle ^k}, \end{aligned}$$

where $z=a,b,c,d$ and $k=2,3,\ldots$. In the special case of $k=2$, which is of particular interest in testing single-PB and single-PIT, the three different types of the boson-number statistics can be considered: the Poissonian [if $g^{(2)}(0)=1$], super-Poissonian [if $g^{(2)}(0)>1$], and sub-Poissonian (otherwise). Analogously, one can define higher-order Poissonian, sub-Poissonian, and super-Poissonian statistics for $k>2$. Such higher-order criteria are not only crucial in analysing multi-PB and multi-PIT effects^11,29,53^, but they are also important in testing whether a specific PB effect is a ‘true’ PB, which can be used for generating single photons or phonons. These higher-order statistics are studied in “Methods”.

**Figure 3:**
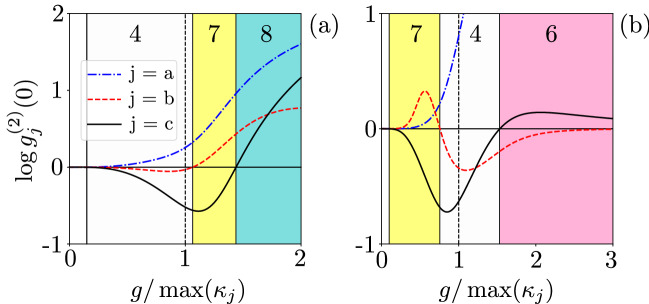
Second-order correlation functions ${g_{i}^{(2)}(0)}$ (in the common logarithmic scale) versus the ratio of qubit-SMR coupling strength and the largest decay rate. Different predictions of the sub- and super-Poissonian boson number statistics, which can be interpreted, respectively, as the PB and PIT effects, of the photonic (a), phononic (b), and hybrid (c) modes assuming: (**a**) the SMR-driven system with parameters specified in Eq. (28) and (**b**) the QD-driven system with Eq. (29). All the shown Cases (i.e., 4, 6, 7, and 8) correspond to those listed in Table 1. The broken line at $g=\max \kappa _j$ is the border line between the strong- and weak-coupling regimes.

Figure 3(a) shows $g^{(2)}(0)$ as a function of the qubit-SMR coupling for the SMR-driven system with the parameters specified in Eq. (28). The regions, when the sub-Poissonian statistics in the hybrid mode *c* is accompanied by the super-Poissonian statistics in the modes *a* and *b*,  are indicated by the yellow background in this and other figures. This area in yellow colour is referred to as Case 7 in Table 1, in which we observe strongly super-Poissonian photons (phonons) in the SMR (QD); whereas a single excitation is observed in the hybrid mode. The system parameters, which lead to Case 7, are found by numerical simulations and are discussed below.

**Table 1:** Different predictions of the super- and sub-Poissonian particle (i.e., photon, phonon or hybrid photon–phonon)-number statistics (PNS) corresponding, respectively, to PIT and PB, for the photon mode *a*, phonon mode *b*, and hybrid photon–phonon mode *c*, where $f_{abc}=\left( \mathrm{sgn}[g_a^{(2)}(0)-1],\mathrm{sgn}[g_b^{(2)}(0)-1],\mathrm{sgn}[g_c^{(2)}(0)-1]\right)$ and the last column indicates each prediction of the mode *a*, *b*, and *c* in the specific colour that is used in our plots.

Case	$f_{abc}$	PNS in mode *a*	PNS in mode *b*	PNS in mode *c*	colour
1	$(-,-,-)$	Sub-Poissonian	Sub-Poissonian	Sub-Poissonian	Aquamarine
2	$(-,-,+)$	Sub-Poissonian	Sub-Poissonian	Super-Poissonian	Lime
3	$(-,+,-)$	Sub-Poissonian	Super-Poissonian	Sub-Poissonian	Light cyan
4	$(+,-,-)$	Super-Poissonian	Sub-Poissonian	Sub-Poissonian	Mint cream
5	$(-,+,+)$	Sub-Poissonian	Super-Poissonian	Super-Poissonian	Plum
6	$(+,-,+)$	Super-Poissonian	Sub-Poissonian	Super-Poissonian	Pink
7	$(+,+,-)$	Super-Poissonian	Super-Poissonian	Sub-Poissonian	Yellow
8	$(+,+,+)$	Super-Poissonian	Super-Poissonian	Super-Poissonian	Cyan

All these cases can be seen in Fig. 10.

Note that Fig. 3a shows these effects in the strong coupling regime^64^, i.e., when the qubit-SMR coupling constant *g* is larger than the system damping rates: $g/\kappa _\mathrm{\max }>1$, where $\kappa _\mathrm{\max }=\max \{\kappa _a, \kappa _b, \gamma \}$. On the other hand, Fig. 3b shows the same yellow region in the weak-coupling regime, i.e., when $g/\kappa _\mathrm{\max }<1$, but this figure was calculated for the QD-driven system, which is discussed in the next section.

By considering the values of Eq. (28), the SMR decay rate is $\kappa _{a}=1.5\gamma$, given that the mode *a* is always in the strong qubit-SMR coupling regime in the region of our interest. This results in Rabi-type oscillations of $g^{(2)}(0)$ that occur in the SMR mode *a* and the hybrid mode *c*. In Fig. 3a, both weak and strong coupling regimes are shown corresponding to *g* smaller or larger than the maximum decay rate of the whole system.

Given the set of parameters in Eq. (28), we are in the good-cavity regime^71^, because $\kappa _{a}<\{\kappa _b,g,f\}$. In the range $g/2\pi \in (4.5,42)$ MHz, the hybrid mode *c* has the sub-Poissonian statistics, while the SMR mode has the super-Poissonian statistics in all the shown cases and a very weak sub-Poissonian statistics occur for phonons in the QD mode *b*, but still corresponding to Case 4 in Table 1. This behaviour changes to the super-Poissonian statistics in the mode b, which corresponds to Case 7, as shown in Fig. 3a. There is a transition for the mode *c* from the sub-Poissonian to super-Poissonian statistics, which corresponds to switching from Case 7 to Case 8 in the strong-coupling regime, where the other two modes are both super-Poissonian. Observing $g^{(2)}(0)>1$ witnesses PIT and the quantum nature of this effect is explored further below.

In order to better probe and understand the dynamics of the system in specific parameter regimes, we analyse also the delay-time second-order photon correlation function, defined as

14 $$\begin{aligned} g_z^{(2)}(\tau )= & {} \lim _{t\rightarrow \infty }\frac{\langle \mathscr{T} : n_z(t+\tau )n_z(t) :\rangle }{\langle n_z(t)\rangle ^2} = \lim _{t\rightarrow \infty }\frac{\langle z^\dagger (t) z^\dagger (t+\tau )z(t+\tau )z(t) \rangle }{\langle z^\dagger (t) z(t)\rangle ^2} , \end{aligned}$$

where $n_z(t)=z^\dagger (t) z(t)$ is the boson number in the modes $z=a,b,c,d$, and the operator products are written in normal order (::) and in time order $\mathscr{T}$. With $g_z^{(2)}(\tau )$ another quantum optical number-correlation phenomenon can be investigated. Specifically, in case of photons, it is referred to as photon antibunching if $g^{(2)}(0)<g^{(2)}(\tau )$, photon unbunching if $g^{(2)}(0)\approx g^{(2)}(\tau )$, and photon bunching if $g^{(2)}(0)>g^{(2)}(\tau )$, which is usually defined for short or very short delay times $\tau$^72^. It is worth noting that photon antibunching was first experimentally observed in the 1970s by Kimble, Dagenais, and Mandel^73^. This was historically the first experimental demonstration of the quantum nature of an electromagnetic field, which cannot be explained classically, unlike photoelectric bunching.

Analogously, one can also investigate the antibunching and bunching of phonons and/or hybrid-mode bosons. Note that the term photon antibunching is often interchangeably used with the sub-Poissonian photon-number statistics^21^. However, to avoid confusion, one can refer to single-time (or zero-delay-time) photon antibunching if defined by $g^{(2)}(0)$ and two-time (or delay-time) photon antibunching if defined via $g^{(2)}(\tau )$.

**Figure 4:**
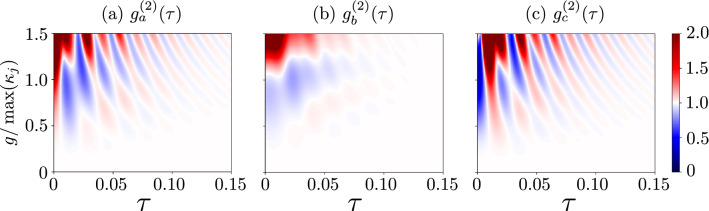
Delay-time second-order correlation functions: (**a**) ${g_{a}^{(2)}(\tau )}$ for the photonic mode, (**b**) ${g_{b}^{(2)}(\tau )}$ for the phononic mode, and (**c**) ${g_{c}^{(2)}(\tau )}$ for the hybrid mode versus the coupling strength $g$ and the delay time $\tau$. We consider here the SMR-driven system with parameters specified in Eq. (28), which enable us to observe the single-photon resonances in the mode *c*. For clarity, all the values of the correlation functions $\ge 2$ are truncated at 2.

**Figure 5:**
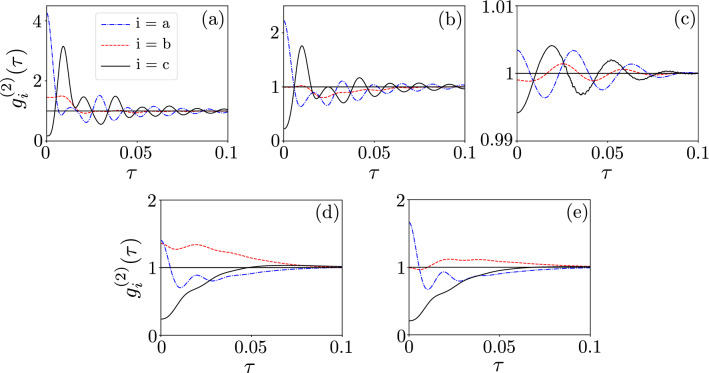
Delay-time second-order correlation functions ${g_{i}^{(2)}(\tau )}$ for the SMR mode *a*, the QD mode *b*, and the hybrid mode *c* modes assuming: (**a**–**c**) the SMR-driven system specified in Eq. (28) with $f=5.5\gamma$ and $\kappa _{\max }=\kappa _b=6\gamma$, and (**d**,**e**) the QD-driven system in Eq. (29) with $\kappa _{\max }=7.5\gamma$, where we additionally set: (**a**) $g=1.3\kappa _{\max }=7.8\gamma$, (**b**) $g=1.1\kappa _{\max }=6.6\gamma$, (**c**) $g=0.2\kappa _{\max }=1.2\gamma$, (**d**) $g=0.7\kappa _{\max }=5.25\gamma$, and (**e**) $g=0.758\kappa _{\max }=5.685\gamma$.

In Fig. 4, we plotted $g^{(2)}(\tau )$ for the range [0, 1.5] of $g/\kappa _{\max }$. This range is also shown in Fig. 3a, where the examples of Cases 4 and 7 can be identified. As expected, one can see oscillations in the SMR and hybrid modes in Figs. 4a,c, respectively. These oscillations are induced by the competition between the qubit-SMR coupling *g* and the SMR-QD hopping *f* in our system. Apparently, by analysing $g^{(2)}(\tau )$ in the weak-coupling regime, the frequency of the oscillations is smaller than that in the strong-coupling regime, in which the oscillations are caused by both couplings *g* and *f*. Moreover in a very weak coupling regime, where $g\ll 1$ oscillations occur due to the hopping strength *f*, with the period $2\pi /f$^74^. This means that, in the weak-coupling regime, also the coupling between the SMR and QD can generate oscillations in our system, where in this case the period of oscillations, which are induced by $f=5.5\gamma$, is approximately equal to $\tau \approx 0.036$, which coincides with the period deduced from the graph, as seen in Fig. 5c. These detrimental oscillations should be suppressed on a time scale longer than the SMR lifetime $\tau =1/\kappa _a$ to enable boson antibunching to survive in the area of our interest.

Various combinations of correlations effects are shown in Fig. 5. All panels in Fig. 5 show that the photon mode *a* is super-Poissonian and bunched, while the hybrid mode *c* is sub-Poissonian and antibunched. However, the properties of the phonon mode *b* are different in every panel. Specifically, the mode *b* is in panel: (a) super-Poissonian and unbunched [defined as $g_b^{(2)}(0)\approx g_b^{(2)}(\tau )$ for non-zero but short delay times $\tau$], (b) Poissonian and unbunched, (c) sub-Poissonian and unbunched, (d) super-Poissonian and bunched, and (e) Poissonian and bunched, as usually considered for very short delay times $\tau$. Note that panels (a, b, c) are for the SMR-driven system, while the remaining panels (d, e) are for the QD-driven system, which are discussed in detail in the next section.

In particular, it is seen that by decreasing the coupling at $g/\kappa _b=1.1$ in Fig. 5b, the QD mode *b* is unbunched with the Poissonian statistics, while the hybrid mode *c* exhibits antibunching $g^{(2)}(0) < g^{(2)}(\tau )$ and the sub-Poissonian statistics $g^{(2)}(0)<1$, in both cases. The role of the auxiliary mode *b* is, in a sense, to convert the super-Poissonian into sub-Poissonian statistics in the mode *c*.

**Figure 6:**
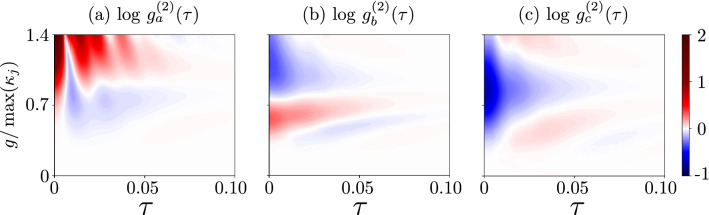
Same as in Fig. 4, but for the QD-driven system with parameters given in Eq. (29). We observe here single-PRs and the corresponding single-PB effects.

**Figure 7:**
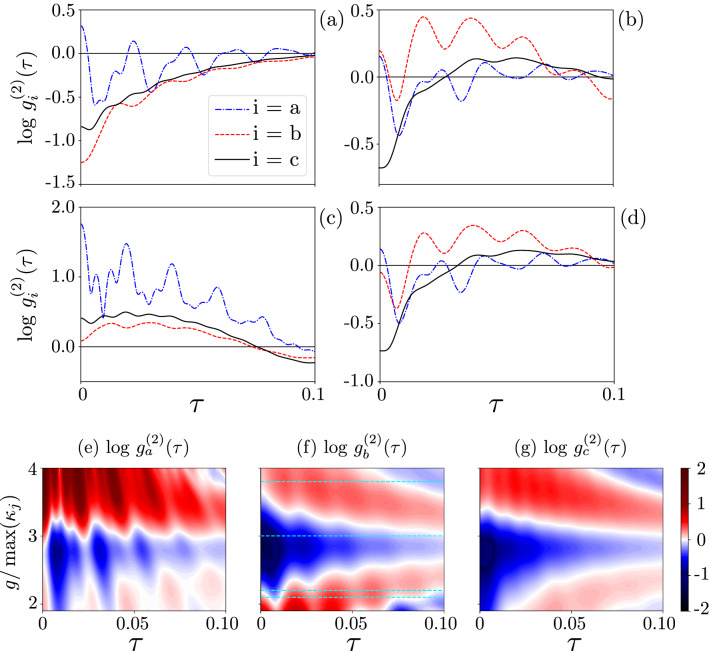
(**a**–**d**) Delay-time second-order correlation functions ${g_{i}^{(2)}(\tau )}$ (in the logarithmic scale) for the SMR mode *a*, the QD mode *b*, and the hybrid mode *c* modes in the QD-driven system assuming that $g/\kappa _{\max }$ is equal to: (**a**) 3, (**b**) 2.1, (**c**) 3.8, and (**d**) 2.2. The four different predictions of correlations for the QD mode *b* correspond to all the cases listed in Table 2. (**e**,**f**) Same as in Fig. 4, but for the parameters given in Eq. (30). Note that panels (**a**–**d**) show the cross-sections of the 3D plot in (**f**) at the values of $g/\kappa _{\max }$ marked by broken lines.

The destructive interference of both modes *a* and *b*, at the balanced linear coupler, can result in the sub-Poissonian statistics of the hybrid modes. We observe this effect even in the weak-nonlinearity (or weak-coupling) regime, which witnesses unconventional PB, as discussed in detail in “Methods”. It is worth noting that in this study we are aiming at observing $g^{(2)}(\tau )<1$ not only at $\tau =0$, but also for non-zero delay times (e.g., $\tau \in [0,0.1]$), as in standard experimental demonstrations of the boson antibunching statistics reported in, e.g., Refs^7,75^. Thus, the cases shown in Fig. 4a,c can hardly be considered as convincing demonstrations of the sub-Poissonian statistics, because of the oscillations, which occur in $g_{a,c}^{(2)}(\tau )$ with increasing $\tau$. More convincing demonstrations of these effects without such oscillations (or by considerably suppressing them) are presented in Figs. 6 and 7, as analysed in detail in the next section.

To explain the super-Poissonian photon-number statistics and photon bunching in the mode *a* for the system pumped in the SMR mode, let us analyse Fig. 5a with $g\approx \kappa _{m}$ concerning the anharmonicity of the energy levels in these cases.

The *g* term in Eq. (2) corresponds to the standard Jaynes–Cummings model with the familiar eigenvalues^62^:

15 $$\begin{aligned} E_n^{\pm } \equiv E(| n,\pm \rangle ) = n\omega _{_\mathrm{SMR}} \pm \frac{1}{2} \sqrt{\Delta _1^2 + \Omega _{n}^2} \end{aligned}$$

with the corresponding eigenstates:

16 $$\begin{aligned} | n,+ \rangle\equiv & {} \cos \left( \tfrac{\theta _n}{2} \right) | n \rangle | e \rangle + \sin \left( \tfrac{\theta _n}{2} \right) | n+1 \rangle | g \rangle , \nonumber \\ | n,- \rangle\equiv & {} - \sin \left( \tfrac{\theta _n}{2} \right) | n \rangle | e \rangle + \cos \left( \tfrac{\theta _n}{2} \right) | n+1 \rangle | g \rangle , \end{aligned}$$

which are often referred to as dressed states or dressed-state dublets, where $\theta _n = \Omega _{n} / \Delta _1$ is the mixing angle, $\Delta _1 = \omega _{q}-\omega _{_\mathrm{SMR}}$ is the detuning between the SMR and qubit. Moreover, $\Omega _{n}=2g\sqrt{n+1}$ can be interpreted as the *n*-photon Rabi frequency on resonance, so, in particular, $\Omega _{0}=2g$ is the vacuum Rabi frequency. Thus, the energy spectrum is clearly anharmonic, which is a necessary condition to observe PB. Note that the Jaynes-Cummings interaction can be effectively described in the dispersive limit (i.e., far off resonance) as a Kerr nonlinearity (for a detailed derivation see, e.g.,^50^), which is the standard nonlinearity assumed in many predictions of PB effects.

To demonstrate the anharmonic energy levels of the complete Hamiltonian $H_{+}$ on resonance (see Fig. 2), we assume a weak drive coupling strength $\eta _{a}$. Given that, the system Hilbert space can be truncated. We assume that the polariton number is at most equal to two in this weak-drive regime. The ground state is $| \psi _0 \rangle =| 0,0,g \rangle$ with the corresponding eigenvalue $E_0=0$. The three eigenvalues of the first manifold (with eigenstates containing a single polariton), as shown in Fig. 2b, are:

17 $$\begin{aligned} E^{(1)}_{1,3}=\Delta \mp \sqrt{g^2+f^2},\quad E^{(1)}_2 =\Delta , \end{aligned}$$

while the five eigenvalues of the second manifold (with eigenstates containing two polaritons), which are shown in Fig. 2c, read:

18 $$\begin{aligned} E^{(2)}_{1,2}&= \frac{1}{2}\left[ 4\Delta -\sqrt{2(3g^2+5f^2\pm f_1)}\right] , \nonumber \\ E^{(2)}_3&= 2\Delta ,\nonumber \\ E^{(2)}_{4,5}&= \frac{1}{2}\left[ 4\Delta +\sqrt{2(3g^2+5f^2\mp f_1)}\right] , \end{aligned}$$

where $f_1=\sqrt{3f^2(10g^2+3f^2)+g^4}$. In particular, by assuming $f=5\gamma$ and $g=7.5\gamma$, the eigenenergies of the first and second manifolds are, respectively: (1) $\Delta$, $\Delta \pm 9.01388\gamma\approx \Delta \pm 9\gamma$, and (2) $2\Delta$, $2\Delta \pm 5.82965\gamma\approx 2\Delta \pm 6\gamma$, and $2\Delta \pm 16.11725\gamma\approx 2\Delta \pm 16\gamma$.

**Figure 8:**
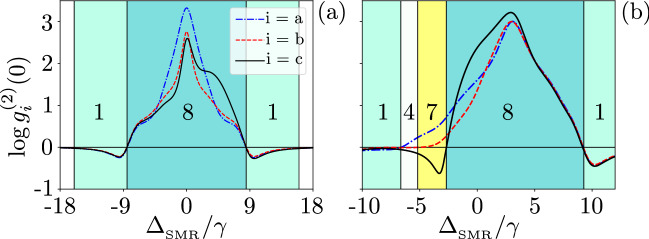
Correlation functions $\log {g_{i}^{(2)}(0)}$ versus the frequency detuning $\Delta _{_\mathrm{SMR}}$ (in units of the qubit decay rate $\gamma$) between the drive and SMR for: (**a**) the resonance case $\omega _{_\mathrm{SMR}}= \omega _{m}=\omega _q$ (so also $\Delta _{_\mathrm{SMR}} =\Delta _{m}=\Delta _q$) and (**b**) the nonresonance case $\omega _{_\mathrm{SMR}}\ne \omega _{m}\ne \omega _q$, where $\omega _b/\gamma =1560$ MHz. Note that by changing the pump frequency, different detunings appear with respect to the modes *a* and *b*, and qubit. We set $g=7.5\gamma$ and other parameters are given in Eq. (28). The numbering of the coloured regions correspond to the cases listed in Table 1.

A simple way to probe the pumped mode is to record the second-order correlation $g^{(2)}(0)$ as a function of $\Delta _{_\mathrm{SMR}}$, where the pump frequency $\omega _p$ is changing (see Fig. 8). To do so, we first consider the resonance case as $\omega _{_\mathrm{SMR}}=\omega _{m}=\omega _q=\omega$ in Eq. (8) and $\omega -\omega _p=\Delta$. As depicted in Fig. 8a, one can see local minima with negative values in $\log g^{(2)}(0)$ for the three modes, which indicate Case 1 in Table 1, at $\Delta _{_\mathrm{SMR}}/\gamma =\pm 9$, which correspond to $\Delta =\pm \sqrt{g^2+f^2}\approx \pm 9\gamma$, given Eq. (17). This means that the pump frequency is located at the two dressed state dublets with energies $E^{(1)}_1$ and $E^{(1)}_3$. And we are off-resonance from the second energy manifold, which implies the possibility of observing PB at these frequencies.

Furthermore, our simulations predict a maximum of $\log g^{(2)}(0)\approx 3$ showing a strong super-Poissonian statistics in the three modes (corresponding to Case 8 in Table 1) as $\Delta _{_\mathrm{SMR}} \rightarrow 0$. In particular, at $\Delta _{_\mathrm{SMR}}/\gamma \approx \pm 6$, the pump frequency is near $E^{(2)}_1\approx 6$ and $E^{(2)}_4\approx -6$, respectively, of the second manifold, in which the probability of the two-photon resonance is maximised, as a signature of PIT. It signifies that the pump is in resonance with one of the levels in the second manifold of the hybrid system energy levels, here specifically $E^{(2)}_1$ and $E^{(2)}_4$. One can see in Fig. 8, peaks (global maxima in the analysed range) of $\log g_n^{(2)}(0)>0$ for $n=a,b,c$ at $\Delta _{_\mathrm{SMR}}=0$. In particular, the probability of absorbing a single photon decreases here. However, if a photon is absorbed, it enhances the probability of capturing subsequent photons, this effect produces the super-Poissonian statistics, which is due to the fact that the probability of observing a single photon is also very small ($P_{10g}\ll 1$) and smaller than the probability of observing two photons^6,76^.

It is seen that, by tuning the drive frequency to the transition $E_2-E_0$ in the energy spectrum of the total nonlinear system, the probability of admitting two photons increases. This results in the super-Poissonian statistics, which is opposite to the case, when the drive frequency is tuned to the transition $E_1-E_0$, when the probability of admitting subsequent photons decreases resulting in PB.

By assuming the off-resonance condition, $\omega _{_\mathrm{SMR}}\ne \omega _{m}\ne \omega _q$, we show in Fig. 8b the correlation functions for the three modes (*a*, *b*, *c*) as a function of $\Delta _{_\mathrm{SMR}}$ in the case, when the drive is tuned in-between the dressed state eigenenergies of the hybrid system.

**Figure 9:**
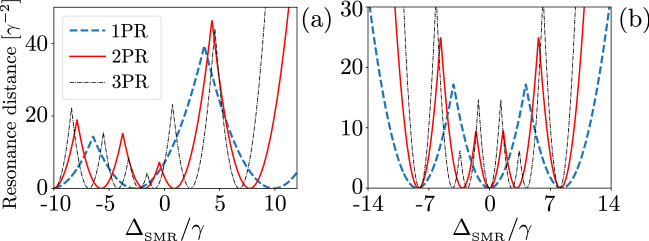
Resonance distances, as defined in Eq. (19), versus the frequency detuning $\Delta _{_\mathrm{SMR}}$ (in units of the qubit decay rate $\gamma$) between the drive and SMR for: (**a**) the SMR-driven system with parameters specified in Eq. (28) with $g=7.58\gamma$ and (**b**) the QD-driven system with Eq. (29) with $g=4.5\gamma$.

The PB and PIT effects observed in Fig. 8 can be explained by considering some measures of the distances from resonances, as shown in Fig. 9a. The distances of the single-, two-, and three-photon resonances (PRs) are defined here, respectively, as:

19 $$\begin{aligned} D_\mathrm{1PR} = \min _i|\omega _p-\omega ^{(1)}_i|^2, \quad D_\mathrm{2PR} = \min _i|2\omega _p-\omega ^{(2)}_i|^2, \quad D_\mathrm{3PR} = \min _i|3\omega _p-\omega ^{(3)}_i|^2, \end{aligned}$$

where $\omega _p$ is the frequency of the pump that is tuned with respect to the energy of the hybrid system. Here $\omega ^{(n)}_i$ are the frequencies (labelled with subscript *i*) in the $n\hbox {th}$ manifold, so the minimalization is performed over $\omega ^{(n)}_i$ for a given manifold *n*. Figure 9 shows the resonance distances versus $\Delta _{_\mathrm{SMR}}$, where $\omega _p$ is tuned with respect to the energy of the whole system. The dip in $g^{(2)}(0)$ at $\Delta _{_\mathrm{SMR}}/\gamma =10$ (see Fig. 8b), which is characteristic for PB, corresponds to the resonance for a single excitation, as seen from $D_\mathrm{1PR}$, and is off-resonance for higher excitations at that frequency (see Fig. 9a). The second-order correlation function $g^{(2)}_c(0)$ for the hybrid mode has a dip as a signature of PB around $\Delta _{_\mathrm{SMR}}/\gamma =-3.4$, while the modes *a* and *b* exhibit the super-Poissonian statistics (indicating PIT), as shown in Fig. 8b. This effect is witnessed as a dip in $D_\mathrm{1PR}$ and it is off-resonance for $D_\mathrm{2PR}$ and $D_\mathrm{3PR}$, as illustrated in Fig. 9a, while the modes *a* and *b* exhibit PIT. This type of unconventional PB is discussed further in sections below.

By decreasing $\Delta _{_\mathrm{SMR}}/\gamma$ from 0 to − 2, the correlation function $g^{(2)}_a(0)$ for the SMR mode in Fig. 8a resembles a shoulder in shape. We observe PIT at this point or region, as expected from our findings in the resonance-distant diagram in Fig. 9a. Indeed, there is a dip in $D_\mathrm{2PR}$ for higher resonances at this point, which explains the occurrence of PIT.

Let us consider now $\Delta _{_\mathrm{SMR}}/\gamma \rightarrow 3$ in Fig. 8b for the pump frequency in resonance with the qubit, $\Delta _q=0$, which is close to the resonance frequency of the hybrid mode. In this case multi-photon transitions are induced, which result in PIT at $\Delta _{_\mathrm{SMR}}/\gamma =3$, and we observe a peak in $\log g^{(2)}(0)>0$ at this frequency in Fig. 8b. Clearly, we are here in resonance with higher-energy levels, while the drive strength is very small, $\eta _{a}/\gamma =0.7$. The probability of observing a single photon is also small as the peak for $\Delta _c= 0$, but if a single photon is absorbed, then the probability of capturing subsequent photons increases, as for PIT.

The analysed system parameters are found by optimising our system to observe the super-Poissonian statistics in the SMR and QD modes. At the sub-Poissonian statistics area of $g^{(2)}(0)$, it is possible to observe in Fig. 14 (in “Methods”) that $g^{(3)}(0)>1$ and/or $g^{(4)}(0)>1$, which are signatures of higher-order photon/phonon resonances and multi-PIT (see “Methods”). Actually, by calculating the second-order correlation function to witness the PB and PIT phenomena, higher-order correlation functions can be used to test whether a given effect is indeed: (1) single-PB or single-PIT, (2) multi-PB or multi-PIT, or (3) nonstandard versions of these effects, as discussed in “Methods” and, e.g., in Refs.^29,53^. As mentioned above, these parameters allow us to achieve the sub-Poissonian statistics for a relatively long delay times.

## Hybrid-mode blockade in the QD-driven system

In this section, we analyse steady-state boson-correlation effects, including the hybrid-mode blockade and PIT, in the QD-driven dissipative system, as described by the Hamiltonian $H''$ and the master equation (12) for the parameters specified mostly in Eqs. (29) and (30).

To eliminate or at least to suppress the undesired oscillations in $g^{(2)}(\tau )$, we assume in this section that our system is driven classically at the QD. Moreover, we assume that the SMR is in the bad-cavity regime, as $\kappa _{_\mathrm{SMR}}\gg g^2/\kappa _{_\mathrm{SMR}} \gg \gamma$^71^. So, we apply the effective system Hamiltonian in the rotating frame, as given by Eq. (9). Even if the lifetime $\tau _{_\mathrm{SMR}}=1/\kappa _{_\mathrm{SMR}}$ of the SMR is much shorter than that assumed in the SMR-driven system, which was discussed in the former section, the hybrid mode, as we show below, reveals no oscillations for quite long delay times, which is due to driving the QD.

To study boson-number statistics of our system, we compute the second-order correlation function $g^{(2)}(0)$ for the optimised parameters, which enables us to demonstrate Cases 4, 6, and 7 of Table 1 in Fig. 3b. In Case 7, which is of our special interest, the modes *a* and *b* are super-Poissonian, as $\log g^{(2)}(0)>0$, while the hybrid mode *c* is sub-Poissonian, as $\log g_c^{(2)}(0)<0$. By increasing the coupling *g* between the SMR and qubit, the mode *b* becomes sub-Poissonian, as being affected by the nonlinearity of the mode *a*.

To check the second criterion for PB, the second-order correlation function $g^{(2)}(\tau )$ is considered below. Figure 6 shows $g^{(2)}(\tau )$ corresponding to $g^{(2)}(0)$ plotted in Fig. 3b showing Cases 4, 6, and 7. As expected, boson antibunching is observed for the hybrid mode, as shown in Fig. 6c, while the SMR mode reveals bunching, as illustrated in Fig. 6a. Moreover both phonon antibunching and bunching, in addition to unbunching [i.e., $g_b^{(2)}(0)\approx g_b^{(2)}(\tau )$ for $\tau \gtrapprox 0$], have been observed in the studied region of the QD mode, as shown in Fig. 6b. It is clear from Fig. 6 that the antibunching of bosons in the three modes survives in some specific coupling regime (around $g=0.7\kappa _m$) for a relatively long delay time $\tau > 1/\kappa$ and oscillations in $g_c^{(2)}(\tau )$ are absent in the hybrid mode *c*. Moreover, boson bunching is observed, when $g_a^{(2)}(\tau )$ drops rapidly for delay times greater than the cavity photon lifetime, as considered in Fig. 5d,e.

**Figure 10:**
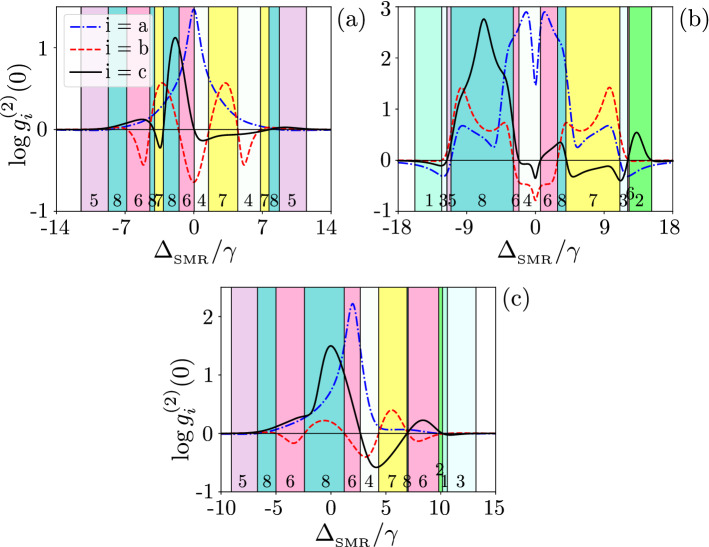
Correlation functions $\log {g_{i}^{(2)}(0)}$ versus the frequency detuning $\Delta _{_\mathrm{SMR}}$ (in units of the qubit decay rate $\gamma$) between the drive and SMR for the QD-driven system for: (**a**,**b**) the resonant case with $\omega _{_\mathrm{SMR}}= \omega _{m}=\omega _q$ (so also $\Delta _{_\mathrm{SMR}}=\Delta _{m}=\Delta _q$), and (**c**) the nonresonant case with $\omega _{_\mathrm{SMR}}\ne \omega _{m}\ne \omega _q$. Parameters are set in: Eq. (29) with $g=4.5\gamma$ for (**a**,**c**), and Eq. (30) with $g=9.5\gamma$ for (**b**). Eight different predictions, which correspond to all the cases listed in Table 1, are marked for the sub- and super-Poissonian number statistics in the photonic (**a**), phononic (**b**), and hybrid photon–phonon (**c**) modes.

To understand the delay-time dependence of the hybrid mode *c*, we consider Eq. (9), when the SMR, QD, and qubit have the same resonance frequency, $\omega _{_\mathrm{SMR}}=\omega _{m}=\omega _q=\omega$ and $g=4.5\gamma$. As illustrated in Fig. 10a, there are three dips (local minima) in $g^{(2)}_b(0)<0$ for the mode *b* of the QD, where we assumed $g<\min \{\kappa _a, \kappa _b\}$ and $f>g$. For these parameters, only a weak nonlinearity is induced in the mode *b*. Thus, the anharmonicity of energy levels cannot explain the PB effect observed as a dip at these three dips (see Fig. 9b). Actually, these dips in $\log g^{(2)}_b(0)$ are due to single-photon resonant transitions, which correspond to unconventional PB, as explained by the non-Hermitian effective Hamiltonian method in the next section and in “Methods”.

Figure 10c shows $\log g_i^{(2)}(0)$ for the three modes as a function of $\Delta _{_\mathrm{SMR}}$. In this case, we assume that the resonance frequencies of the SMR, QD, and qubit are not the same, and the detuning of each mode with respect to $\omega _p$ is different. It is shown that, when $\Delta _{_\mathrm{SMR}}/\gamma \rightarrow 2$, multiphoton transitions (and so PIT or multi-PB) can be induced in the mode *a*, where the pump frequency is in the resonance with the qubit, $\omega _p=\omega _q$. This effect is seen in Fig. 14 (in “Methods”) corresponding to a local maximum in higher-order moments $g_{i}^{(3)}(0)$ and $g_{i}^{(4)}(0)$. Likewise the resonance case, unconventional PB in the modes *b* and *c* can be explained by the method applied in the next section.

**Figure 11:**
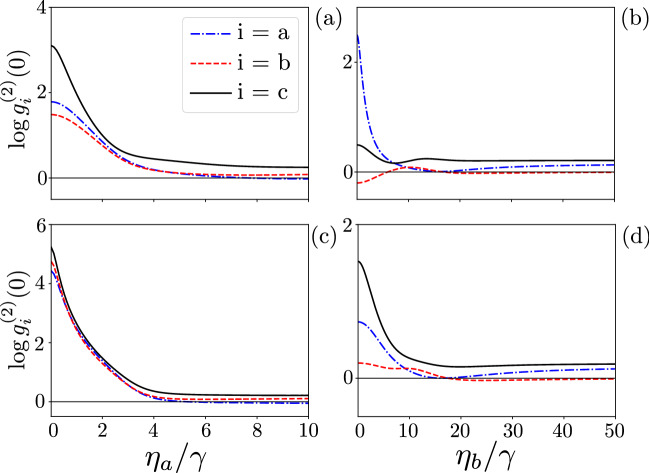
Second-order correlation functions $\log {g_{i}^{(2)}(0)}$ versus the drive strengths: (**a**,**c**) $\eta _{a}$ for the SMR-driven system and (**b**,**d**) $\eta _{b}$ for the QD-driven system. Parameters are given in: (**a**) Eq. (28) with $g=7.5\gamma$ and $\omega _p=1554\gamma$, which implies $\Delta _q=-3\gamma$, $\Delta _b=6\gamma$, $\Delta _a=0$; (**b**) Eq. (29) with $\omega _p=1568\gamma$, which implies $\Delta _q=0$, $\Delta _b=-8\gamma$, and $\Delta _a=2\gamma$; (**c**) Eq. (28) with $g=7.5\gamma$ and $\omega _p=1551\gamma$, which implies $\Delta _q=0$, $\Delta _b=9\gamma$, and $\Delta _a=3\gamma$; and (**d**) Eq. (29) with $\omega _p=1570\gamma$, which implies $\Delta _q=-2\gamma$, $\Delta _b=-10\gamma$, and $\Delta _a=0$.

In Fig. 11, we study how the second-order correlation functions reveal the PIT regime, which corresponds to Case 8 in Table 1, as a function of the SMR-pump strength $\eta _a$ [in panels (a) and (c)] and the QD-pump strength $\eta _b$ [in panels (b) and (d)]. The hybrid mode *c* is super-Poissonian for all the shown cases and pump strengths. The modes *a* and *b* are super-Poissonian [except the mode *a* in panel (b)] for small pump strengths $\eta _{a,b}$. By increasing the driving power at least to some values, which can be identified in the figures for specific modes, we observe that the correlation functions $g^{(2)}(0)$ also decrease for all the modes (except the mentioned case). This property confirms the nonclassicality of the predicted PIT in the hybrid system according to an additional criterion of ‘true’ PIT of Ref.^14^.

## Unconventional blockade explanation via non-Hermitian Hamiltonian approach

In this section, we apply the analytical mathematical formalism of Ref.^45^, based on an non-Hermitian Hamiltonian, to identify the quantum interference effect that is responsible for inducing unconventional PB, i.e., strongly sub-Poissonian statistics in the weak-coupling regime or the weak-nonlinearity regime. We stress that this is an approximate approach, where the effect of quantum jumps is ignored^77,78^.

By considering the system studied in the former section under the weak-pump condition, we can truncate the Hilbert spaces for the modes *a* and *b* and the qubit at their two excitations in total. This allows us to consider the total-system Hilbert space of dimension $3\times 3\times 2=18$. Moreover, the weak-pump condition implies that $C_{00g}\gg C_{10g}, C_{01g}, C_{00e}\gg C_{11g}, C_{10e}, C_{01e}, C_{20g}, C_{02g}$. Thus, the steady-state of the coupled system can be expressed as

20 $$\begin{aligned} | \Psi _{abq}(t) \rangle&= C_{00g}| 00g \rangle +e^{-i\omega _d t} \left( C_{00e}| 00e \rangle +C_{10g}| 10g \rangle +C_{01g}| 01g \rangle \right) \nonumber \\&\quad +e^{-2 i \omega _d t} \left( C_{10e}| 10e \rangle +C_{01e}| 01e \rangle +C_{11g}| 11g \rangle +C_{20g}| 20g \rangle +C_{02g}| 02g \rangle \right) , \end{aligned}$$

where $| n_a,n_b,g/e \rangle$ is the Fock state with $n_a$ photons in the SMR, $n_b$ phonons in the QD, and the lower ($| g \rangle$) or upper ($| e \rangle$) state of the qubit. The effective non-Hermitian Hamiltonian of the system can be written as

21 $$\begin{aligned} H_\mathrm{eff}= H''-i\frac{\kappa _a}{2}a^\dagger a-i\frac{\kappa _b}{2}b^\dagger b -i\frac{\gamma }{2}\sigma _{+} \sigma _{-}, \end{aligned}$$

where $H''$ is given by Eq. (9). Analogously, one can consider the non-Hermitian Hamiltonian with $H'$, given by Eq. (8).

In the weak-pump regime, the mean number of photons and phonons in the SMR and QD can be approximated as $\langle n_a\rangle \approx | C_{10g}|^2$ and $\langle n_b\rangle \approx | C_{01g}|^2$, respectively. As derived in detail in “Methods”, the second-order correlation functions for generated photons and phonons, under the same weak-pump conditions, can be given by:

22 $$\begin{aligned} g^{(2)}_a(0)&= \frac{\langle a^\dagger a^\dagger a a \rangle }{\langle a^\dagger a\rangle ^2}\approx \frac{2| C_{20g}|^2}{| C_{10g}|^4},\nonumber \\ g^{(2)}_b(0)&= \frac{\langle b^\dagger b^\dagger b b\rangle }{\langle b^\dagger b\rangle ^2}\approx \frac{2| C_{02g}|^2}{| C_{01g}|^4}, \end{aligned}$$

where the superposition coefficients $C_{n,m,g}$ are given in Eqs. (39) and (41).

The hybrid photon–phonon modes, which are defined in Eq. (10), are the output modes of the balanced linear coupler with the SMR and QD modes at its inputs. As shown in “Methods”, we find, analogously to Eq. (22), the second-order correlation function for the hybrid mode *c* reads:

23 $$\begin{aligned} g^{(2)}_c(0)= \frac{\langle c^\dagger c^\dagger c c \rangle }{\langle c^\dagger c\rangle ^2}\approx \frac{2| C^\prime _{20g}|^2}{| C^\prime _{10g}|^4}, \end{aligned}$$

where the superposition coefficients $C'_{n,m,e/g}$ are given in Eqs. (40) and (41), and the sixth formula in Eq. (34).

This approach enables us to explain unconventional PB generated in the hybrid system, which is the result of a destructive quantum interference effect that assures, together with other conditions, that the probability amplitude of having two photons in the SMR and QD is negligible. This method can also be used to find some optimal parameters to observe PB in the system.

**Figure 12:**
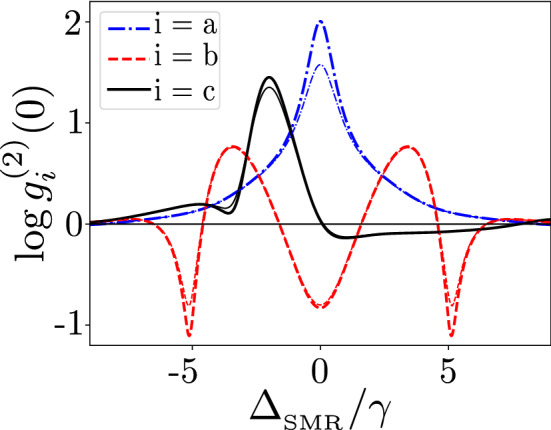
Correlation functions $\log {g_{i}^{(2)}(0)}$ versus the frequency detuning $\Delta _{_\mathrm{SMR}}$ in units of $\gamma$ for the QD-driven system for the resonant case with $\omega _{_\mathrm{SMR}}=\omega _{m}=\omega _q=\gamma \times 1560$ MHz (so also $\Delta _a=\Delta _b=\Delta _q$). The thin curves in each mode are obtained using the master equation in Eq. (12) and the thick curves are obtained from the non-Hermitian Hamiltonian method using Eqs. (22) and (23). Parameters are set in Eq. (29) except $g=4.5\gamma$ and $\kappa _a=\kappa _b=6\gamma$.

Figure 12 presents a comparison of our predictions based on the precise numerical solutions of the master equation in Eq. (12), as shown by thin curves, with those calculated from Eqs. (22) and (23) using the non-Hermitian Hamiltonian approach, as shown by thick curves. The locations of the maxima and minima of the correlation functions are found similar according to both formalisms. However, these extremal values can differ more distinctly, especially for the two global minima in the sub-Poissonian statistics of the mode *b* and the super-Poissonian maximum of the mode *a*. The differences result from the effect of quantum jumps, which are properly included in the master-equation approach and totally ignored in the non-Hermitian Hamiltonian approach (Fig. 12).

**Table 2:** Different single- and two-time phonon-number correlation effects induced in the QD mode, which can be observed for different values of the qubit-SMR coupling strength *g* with respect to the SMR decay rate $\kappa _{a}$, e.g., by setting the other parameters to be the same as in Eq. (30).

Case	Effect	Single-time correlations	Two-time correlations	Example of $g/\kappa _{a}$	Figure
I	Stronger form of PB (‘true’ PB)	Sub-Poissonian PNS ${g_{b}^{(2)}(0)}<1$	Phonon antibunching ${g_{b}^{(2)}(\tau )} >{g_{b}^{(2)}(0)}$	3.0	7(a)
II	Stronger form of PIT (‘true’ PIT)	Super-Poissonian PNS ${g_{b}^{(2)}(0)}>1$	Phonon bunching ${g_{b}^{(2)}(\tau )} <{g_{b}^{(2)}(0)}$	2.1	7(b)
III	Weaker form of PIT or PB	Super-Poissonian PNS ${g_{b}^{(2)}(0)}>1$	Phonon antibunching ${g_{b}^{(2)}(\tau )} >{g_{b}^{(2)}(0)}$	3.8	7(c)
IV	Weaker form of PB or PIT	Sub-Poissonian PNS ${g_{b}^{(2)}(0)}<1$	Phonon bunching ${g_{b}^{(2)}(\tau )} <{g_{b}^{(2)}(0)}$	2.2	7(d)

Here, PNS stands specifically for the phonon-number statistics of the mode *b*. Note that we also found examples of Cases I, II, and IV for the modes *a* and *c* using the same system parameters as for the mode *b*.

## Different types of blockade and tunnelling effects

The sub-Poissonian statistics of a bosonic field, as described by $g^{(2)}(0)\ll 1$, is not a sufficient criterion for observing a ‘true’ PB, which can be a good single-photon or single-phonon source. In fact, other criteria, such boson antibunching, $g^{(2)}(0)< g^{(2)}(\tau )$, and the sub-Poissonian statistics of higher-order correlation functions, $g^{(n)}(0)\ll 1$, should also be satisfied (see “Methods”). Anyway, most of the studies of PB, and especially those on unconventional PB, are limited to testing the second-order sub-Poissonian statistics described by $g^{(2)}(0)< 1$.

As explicitly discussed in Refs.^21,72,79,80^, photon antibunching and sub-Poissonian statistics are different photon-number correlation effects. So, the four cases listed in Table 2, can be considered as different types of PB and PIT. We show that all these effects can be observed in the studied system. For brevity, Table 2 is limited to phononic effects. PB, as defined in Case I and often referred to as a ‘true’ PB, can be a good single-photon sources; but, as mentioned above, other higher-order criteria should also be satisfied.

To show these four different effects, we use the parameters set in Eq. (30), where $\kappa _{b} \ll \kappa _{a}$ at the $\kappa _b=0.002\, \gamma$, which indicates that the quality factor is $Q\approx 200,$ and so $\eta _{b}/\kappa _{b}\approx 100$ in the case of a strong pump driving the QD mode with $\eta _{b}=0.22\, \gamma$. Apart from the previously mentioned phenomena, such as observing the super-Poissonian statistics and bunching in the SMR and QD modes, while a hybrid mode exhibiting the sub-Poissonian statistics and boson antibunching, we find the four types of PB/PIT in the mode *b* in different coupling regimes, as shown in Table 2, which includes the examples of specific experimentally feasible values of $g/\kappa _{a}$.

Case I corresponds to a stronger form of PB, which we refer to as a ‘true’ PB, when the nonclassical nature of bosons is revealed by both their antibunching and sub-Poissonian statistics. Case II corresponds to a stronger form of PIT, which can be called a ‘true’ PIT, when bosons exhibit both classical effects: the super-Poissonian statistics and bunching. In Case III, one can talk about a weaker form of PIT or, equivalently, another weaker type of PB, as such bosons are characterised by the classical super-Poissonian statistics and their nonclassical nature is revealed by antibunching. Case IV represents another weaker form of PB or, equivalently, of PIT, which is characterised by the nonclassical sub-Poissonian statistics of classically bunched bosons. These results imply that one cannot say in general that the antibunching of bosons leads to their sub-Poissonian statistics and vice versa^21,79^.

Therefore, $g^{(2)}(\tau )>g^{(2)}(0)$ does not necessarily imply $g^{(2)}(0)<1,$ as in Case III, which can be seen in Fig. 7c,f. In addition, as another example related to Case IV, let us consider a Fock state $| n \rangle$ with $n\ge 2$, for which $g^{(2)}(0)=1-1/n$, such that if $n=2$ then $g^{(2)}(0)=0.5,$ so $g^{(2)}(0)<1$ and it is not accompanied by boson antibunching, but bunching in this case.

Our focus in this paper is on the generation of PB in the hybrid mode, while the other two modes exhibit PIT. Note that this a very special case of Table 1, which shows that eight combinations of boson number correlation phenomena in the modes *a*, *b*, and *c* can be generated in our system, as specified by the numbered coloured regions in various figures corresponding to the cases in Table 1. Thus, we found all the eight possible combinations of the PIT and PB effects in the hybrid system for the parameters specified in Eqs. (28), (29), and (30).

## Detection of the hybrid-mode correlation functions

**Figure 13:**
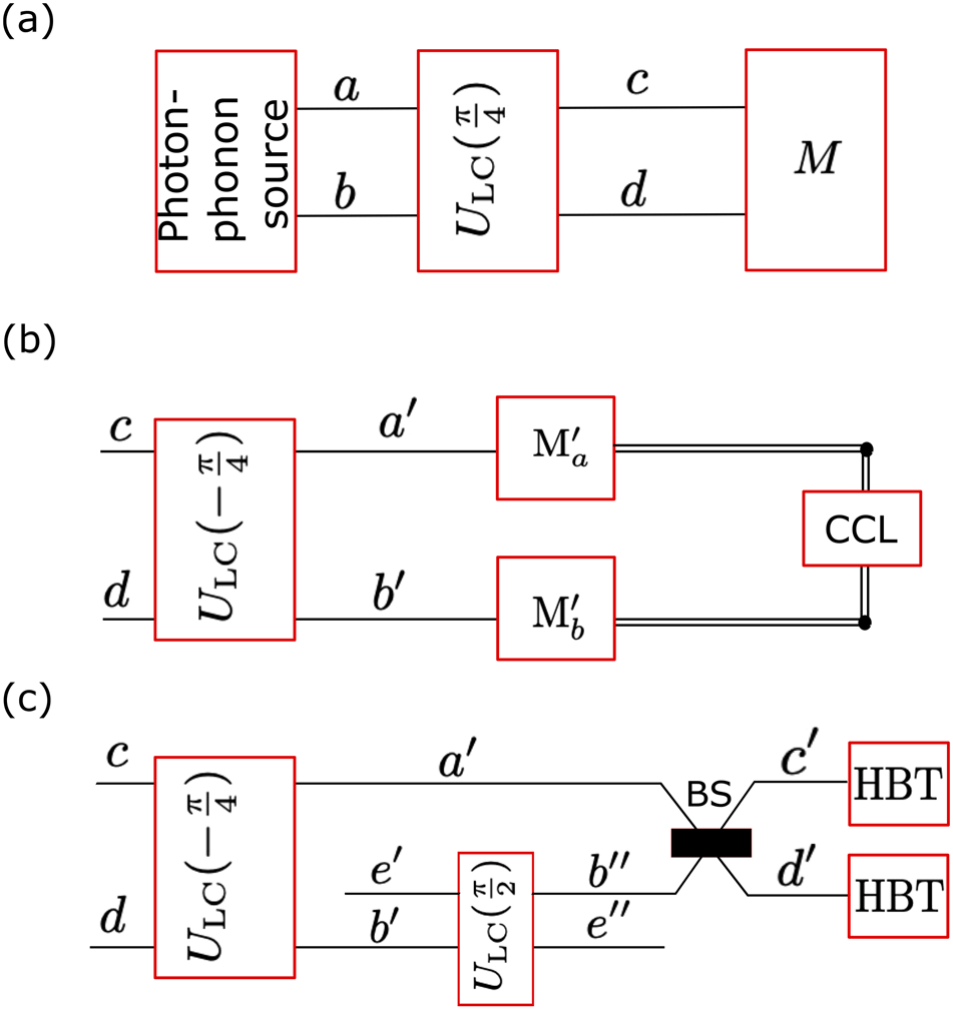
Schematics of the proposed detection schemes: (**a**) General scheme for the generation of the photonic mode *a*, phononic mode *b*, and hybrid modes *c*, *d*, and their detection in the measurement unit *M*, which is shown in specific implementations using: (**b**) detection method 1 and (**c**) detection method 2. Key: $U_\mathrm{LC}(\theta )$ stands for the linear-coupler transformation, which in special cases corresponds to multi-level SWAP (for $\theta =\pi /2$) and Hadamard-like (for $\theta =\pm \pi /4$) gates; BS is the balanced beam splitter, which corresponds to $U_\mathrm{LC}(\pi /4)$, $M'_a$ ($M'_b$) is a measurement unit for detecting photons (phonons), CCL is a coincidence and count logic unit, HBT stands for the standard Hanbury–Brown and Twiss optical interferometer. Mode $e'$ ($e''$) is in the photonic (phononic) vacuum state.

Here, we describe two detection schemes for measuring the intensity autocorrelation functions for the hybrid photon–phonon modes *c* and *d*, as shown in Fig. 13.

The measurements of $g^{2}(\tau )$ for the photonic mode *a* and the phononic mode *b* are quite standard and are usually based on the Hanbury-Brown and Twiss (HBT) optical interferometry and its generalised version for phonons^81^, respectively. However, the measurement *M* (as schematically shown in Fig. 13a) of $g^{(2)}(\tau )$, or even $g^{(2)}(0)$, for the hybrid photonic-phononic modes *c* and *d* is quite challenging if applied directly. Here we propose two detection methods, as shown in Fig. 13b,c, for indirect measuring of $g_{c,d}^{(2)}(0)$.

The first operation of the measurement unit *M* in both schemes is a linear-coupler transformation of the hybrid modes (*c*, *d*) into $(a',b')$, which, assuming that the process is perfect, should be equal to the original purely photonic (*a*) and phononic (*b*) modes.

We consider a linear coupler (formally equivalent to a beam splitter) described by a unitary operation $U_\mathrm{LC}(\theta )$, which transforms the input operators *a* and *b* into:

24 $$\begin{aligned} c(\theta )&= U^\dagger _\mathrm{LC}(\theta ) a U_\mathrm{LC}(\theta ) = a \sin \theta + b \cos \theta , \nonumber \\ d(\theta )&= U^\dagger _\mathrm{LC}(\theta ) b U_\mathrm{LC}(\theta ) = a \cos \theta - b \sin \theta , \end{aligned}$$

for a real parameter $\theta$, where $T=\cos ^2\theta$ and $R=1-T=\sin ^2\theta$ are the transmission and reflection coefficients of the linear coupler, respectively. The studied hybrid modes are the special cases of Eq. (24) for $c\equiv c(\theta =\pi /4)$ and $d\equiv d(\theta =\pi /4)$. Clearly, the first transformation $U_\mathrm{LC}(-\pi /4)$ in Fig. 13b,c, is the transformation inverse to that in Fig. 13a.

### Detection method 1 based on measuring photons and phonons

The correlation functions $g_{c,d}^{(2)}(0)$ in the hybrid photon–phonon modes can be measured indirectly, as indicated in Fig. 13b, by measuring the observables:

25 $$\begin{aligned} f_{kl}=(a^\dagger )^k a^l,\quad g_{mn}=(b^\dagger )^m b^n, \end{aligned}$$

where $k,l,m,n=0,1,2$, by using the relations:

26 $$\begin{aligned} \langle c^{\dagger } c \rangle= \frac{1}{2} \left( \langle f_{11} \rangle + \langle g_{11} \rangle + \langle f_{01}g_{10} \rangle + \langle f_{10}g_{01} \rangle \right) , \end{aligned}$$

27 $$\begin{aligned} \langle c^{\dagger 2} c^2 \rangle = \frac{1}{4} \left( \langle f_{22} \rangle + 4\langle f_{11}g_{11} \rangle + \langle g_{22} \rangle + 2\langle f_{01}g_{21} \rangle + 2\langle f_{10}g_{12} \rangle + \langle f_{20}g_{02} \rangle +\langle f_{02}g_{20} \rangle + 2\langle f_{21}g_{01} \rangle + 2\langle f_{12}g_{10} \rangle \right) , \end{aligned}$$

and analogous relations for the hybrid mode *d*. The measurement units $M'_a$ and $M'_b$ in this method, as shown in Fig. 13b, describe the measurements of photons and phonons, respectively. It is seen that, in this approach, to determine $g_{c,d}^{(2)}(0)$, one has to measure the following observables: $f_{01}$, $f_{10}$, $f_{11}$, $f_{02}$, $f_{20}$, $f_{12}$, $f_{21}$, and $f_{22}$. Almost each observable $f_{kl}$ should be measured simultaneously with a specific observable $g_{mn}$, which can be realised by a coincidence and count logic (CCL) unit in Fig. 13b.

The measurements of all the required photonic observables $f_{kl}$ can be performed by using, e.g., the Shchukin–Vogel method, which is based on balanced homodyne correlation measurements^82^. According to that method, a photonic signal is superimposed on a balanced beam splitter with a local oscillator, which is in a coherent state $| \alpha =|\alpha |\exp (\phi ) \rangle$ with a tunable phase $\phi$. A desired mean value of the observable $f_{kl}$ can be obtained by linear combinations of the coincidence counts registered by specific detectors for different local-oscillator phases $\phi$. This part of the method corresponds to a Fourier transform. The simplest nontrivial configuration, which enables the measurement of the observables $f_{10}$, $f_{01}$, $f_{20}$, and $f_{02}$, requires four detectors and three balanced BSs, where additional input ports are left empty, i.e., allowing only for the quantum vacuum noise. By replacing the four detectors with four balanced BSs with altogether eight detectors at their outputs, one can measure any observable $f_{kl}$ for $k+l\le 4$. These include the desired observables $f_{21}$, $f_{12}$, and $f_{22}$. Of course, the observable $f_{22}$ can be measured in a simpler way via the HBT interferometry. The measurement of phononic observable $g_{mn}$ can be performed analogously just by replacing the balanced BSs by balanced phonon-mode linear couplers and using phonon detectors as, e.g., in Ref.^81^. The measurement of two-mode moments $\langle f_{kl}g_{mn} \rangle$ is, at least conceptually, a simple generalisation of the single-mode methods relying on proper coincidences in photonic and phononic detectors. Note that a multimode optical version of the original single-mode method was described in Ref.^83^.

### Detection method 2 based on measuring only photons

Figure 13c shows another realisation of the measurement unit *M*, to determine $g_{c,d}^{(2)}(0)$, and even $g_{c,d}^{(2)}(\tau )$. This method is, arguably, simpler and more effective than detection method 1, because it is based on measuring only photons and using standard HBT interferometry. Our approach was inspired by Ref.^48^, where the measurement of single-mode phonon blockade was described via an optical method instead of a magnetomotive technique, which was described in Ref.^47^, where phonon blockade was first predicted.

Our measurement setup realises the following three transformations: (1) converting the phononic mode $b'$ into a photonic mode $b''$, (2) mixing the optical modes $a'$ and $b''$ on a balanced BS to generate the modes $c'$ and $d'$, which, in an ideal case, have the same boson-number statistics as the original hybrid photon–phonon modes *c* and *d*; and finally, (3) applying the conventional optical HBT interferometry for these two optical modes. In unit (1), this conversion corresponds to a multi-level SWAP gate, which can be implemented by a photonic–phononic linear coupler for $\theta =\pi /2,$ assuming that the auxiliary input mode $e'$ is in the photonic vacuum state, while the output mode $e''$ is in the phononic vacuum state. In unit (3), the balanced BS action on the optical modes $a'$ and $b''$ in Fig. 13c corresponds to the transformation of the balanced linear coupler on the photonic (*a*) and phononic (*b*) modes, as shown in Fig. 13a.

Clearly, the linear-coupler transformation $U_\mathrm{LC}(\theta )$ is applied not only to the modes (*a*, *b*), but also to other modes. Thus, Eq. (24) should be adequately modified by replacing (*a*, *b*) by (*c*, *d*), $(e',b')$, and $(a',b'')$. For brevity, we omit their explicit obvious definitions here. Note that $U_\mathrm{LC}(\pi /2)$ and $U_\mathrm{LC}(\pi /4)$ correspond to a multi-level SWAP and Hadamard-like gates, respectively; while the balanced BS in Fig. 13c corresponds to $U_\mathrm{LC}(\pi /4)$.

## Discussion

We proposed a novel type of boson blockade, as referred to as hybrid photon–phonon blockade, which is a generalisation of the standard photon and phonon blockade effects. We predicted the new effect in a hybrid mode obtained by linear coupling of photonic and phononic modes. We described how hybrid photon–phonon blockade can be generated and detected in a driven nonlinear optomechanical superconducting system. Specifically, we considered the system composed of linearly coupled microwave and mechanical resonators with a superconducting qubit inserted in one of them.

We studied boson-number correlations in the photon, phonon, and hybrid modes in the system. By analysing steady-state second-order correlation functions, we found such parameter regimes of the system for which four different types of boson blockade and/or boson-induced tunnelling can be observed. Thus, we showed that bosons generated in the studied system can exhibit the sub-Poissonian (or super-Poissonian) boson-number statistics accompanied by boson antibunching in some cases or bunching in others. These results can be interpreted as four different types of blockade or tunnelling effects, as summarised in Table 2.

By tuning the pump frequency with respect to the energy levels of the hybrid system, which is driven via the SMR, we showed that it is possible to observe PB and PIT that can be explained by a large energy-level anharmonicity in the strong-coupling (or large-nonlinearity) regime. However, the time evolution of the second-order correlation function $g^{(2)}(\tau )$ oscillates due to the coupling *g* between the SMR and qubit as well as the hopping *f* between the SMR and QD. We showed that it is possible to induce PB in the hybrid mode *c* that survives for much longer delay times by driving the QD instead of the SMR.

We also predicted unconventional PB in the three modes in the weak-coupling (or weak-nonlinearity) regime using a non-Hermitian Hamiltonian approach based on neglecting quantum jumps. Our analytical approximate predictions are in a relatively good agreement with our precise master-equation solutions (including quantum jumps).

Moreover, as summarised in Table 1, we showed the possibility to observe eight different combinations of either PB or PIT in the three modes (*a*, *b*, and *c*) in different coupling regimes of this system. Thus, in particular, we found that the tunnelling effects in the photonic and phononic modes can lead, by their simple linear mixing, to the hybrid photon–phonon blockade effect.

Finally, we discussed two methods of detecting hybrid-mode correlations. One of them is based on measuring various moments of photons and phonons via balanced homodyne correlation measurements. While the other method is based on converting phonons of the hybrid mode into photons, by using a linear coupler acting as a multi-level SWAP gate, and then applying the standard optical HBT interferometry.

We believe that our study of the interplay between photons and phonons can lead to developing new experimental methods for controlling and testing the quantum states of mechanical systems with atom-cavity-mechanics polaritons. We hope that our work can also stimulate research on quantum engineering with hybrid photon–phonon modes.

## Methods

### Parameters used in our simulations

Our figures, as indicated in their captions, are plotted for the SMR-driven dissipative system described by the Hamiltonian $H'$, given in Eq. (8), assuming:

28 $$\begin{aligned} A_1= \{\Delta _a=-3\gamma , \Delta _b=3\gamma , \Delta _q = -6\gamma , f=5\gamma , \eta _a= 0.7\gamma , \eta _b= 0, \kappa _a=1.5\gamma , \kappa _b=6\gamma \}, \end{aligned}$$

and for the QD-driven dissipative system for the Hamiltonian $H''$, given in Eq. (9), assuming either

29 $$\begin{aligned} A_2= \{ \Delta _a=5\gamma , \Delta _b=-5\gamma , \Delta _q = 3\gamma , f=7\gamma , \eta _a= 0, \eta _b= 0.5\gamma , \kappa _a=7.5\gamma , \kappa _b=6\gamma \}, \end{aligned}$$

or

30 $$\begin{aligned} A_3= \{\Delta _a=4\gamma , \Delta _b=-4\gamma , \Delta _q = 7\gamma , f=6.4\gamma , \eta _a= 0, \eta _b= 0.22\gamma , \kappa _a=3.5\gamma , \kappa _b=0.002\gamma \},\quad \end{aligned}$$

where $\gamma =10\pi$ MHz. Minor modifications of these parameters are specified in figure captions.

### Higher-order correlation effects

Here we briefly study the $k\hbox {th}$-order boson-number correlation functions $g_z^{(k)}(0)$, as defined in Eq. (13) for $k=3,4,$ in comparison to the standard second-order function $g_z^{(2)}(0)$ for the photon ($z=a$), phonon (*b*), and hybrid photon–phonon (*c*) modes.

**Figure 14:**
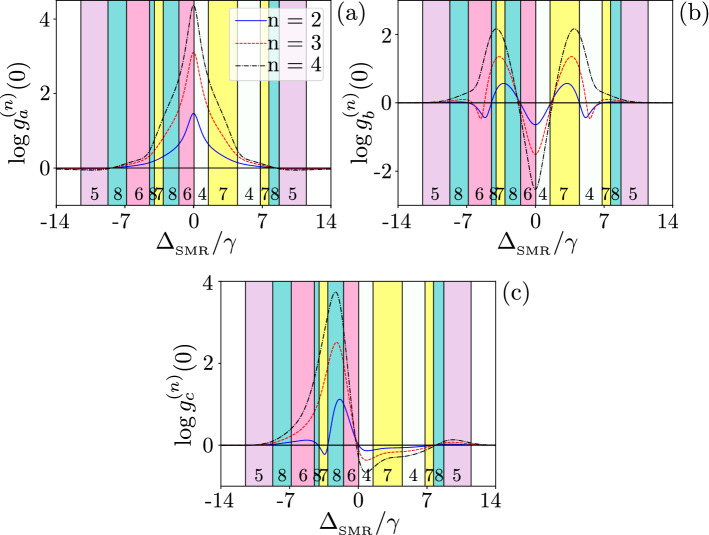
Correlation functions $\log g_{i}^{(n)}(0)$ of various orders [second (solid curves), third (dashed), and forth (dot-dashed)] versus the detuning between the drive and SMR (in units of the qubit decay rate $\gamma$) for the QD-driven system for: (**a**) the photonic mode *a*, (**b**) the phononic mode *b*, and (**c**) the hybrid mode *c*. All parameters and colourful regions are the same as in Fig. 10a.

Figure 14 shows our results for $g_z^{(3)}(0)$ (dashed curves) and $g_z^{(4)}(0)$ (dot-dashed curves) in comparison to $g_z^{(2)}(0)$ (solid curves) for $z=a,b,c$ in corresponding panels. Note that the same curves for $g_z^{(2)}(0)$ are also shown in Fig.  10a, but we repeat them for a better comparison with $g_z^{(3,4)}(0)$. It is seen that the eight cases of Table 1 can be divided into a number of subcases depending on $g_z^{(3)}(0)$ and $g_z^{(4)}(0)$. Such a classification is quite complex as includes, in principle, $8^3=512$ cases. So, instead of that, we present another much-simplified classification of eight cases only, as shown in Table 3 using the auxiliary function $g_{234}$ defined as:

31 $$\begin{aligned} g_{234} = \left[ \mathrm{sgn}\log g_z^{(2)}(0),\mathrm{sgn}\log g_z^{(3)}(0),\mathrm{sgn}\log g_z^{(4)}(0)\right] . \end{aligned}$$

In particular $[-,-,-]$ means that the second-, third- and fourth-order sub-Poissonian photon number-statistics are observed in a given mode, which are the necessary conditions for observing a ‘true’ single-PB. This case can be easily identified in both panels of Fig. 14. One can also find the case when $[+,+,+]$, which corresponds to the super-Poissonian statistics of orders $k=2$, 3, and 4, which might be interpreted, as the induced tunnelling by one, two, and three photons. However, we can also find intermediate four out of six cases, which can be interpreted as non-standard types single-PB and/or single-PIT, and in some cases can be identified as multi-PB^11,26,29,30,53^. However, a detailed classification of such multi-PB and their interpretation is not at the focus of this paper. The presented results show only the possibility of generating in our system a plethora of various photon–phonon correlation effects, which can be revealed by higher-order correlation functions for the experimentally feasible parameters.

**Table 3:** Different predictions of the *n*th-order super- and sub-Poissonian statistics with $n=2,3,4$ for the photon ($z=a$), phonon (*b*), and hybrid photon–phonon (*c*) modes, where $g_{234}$ is defined in Eq. (31).

Case	$g_{234}$	Mode *a*	Mode *b*	Mode *c*
1	$(-,-,-)$	$\surd$	$\surd$	$\surd$
2	$(-,-,+)$	$\times$	$\surd$	$\surd ^*$
3	$(-,+,-)$	$\times$	$\times$	$\times$
4	$(+,-,-)$	$\surd$	$\surd$	$\surd$
5	$(-,+,+)$	$\times$	$\surd$	$\surd$
6	$(+,-,+)$	$\times$	$\times$	$\times$
7	$(+,+,-)$	$\surd$	$\surd$	$\surd$
8	$(+,+,+)$	$\surd$	$\surd$	$\surd$

The cases marked with $\surd$ can be identified under both (1) nonresonance conditions, as shown in Fig. 14b, and (2) resonance conditions, as shown in Fig. 14a, except the case marked with $^*$.

### Analytical approach via non-Hermitian Hamiltonian in Eq. (21)

Here, we follow the method of Ref.^45^ to derive the coefficients $C_{n,m,k}$ and $C^\prime _{n,m,k}$ for $n,m \in {0,1,2}$ and $k=e,g$, which appear in Eqs. (22) and (23).

First we recall that the balanced linear coupler (or a balanced beam splitter) transformation, which leads to Eq. (24), if applied to the input Fock states $| n_a, n_b \rangle$ for $n_a+n_b\le 2$ yields:

32 $$\begin{aligned} | 10 \rangle \rightarrow \frac{1}{\sqrt{2}}(| 10 \rangle -| 01 \rangle ), \quad | 01 \rangle \rightarrow \frac{1}{\sqrt{2}}(| 10 \rangle +| 01 \rangle ), \quad | 11 \rangle \rightarrow \frac{1}{\sqrt{2}}(| 20 \rangle -| 02 \rangle ),\nonumber \\ | 02 \rangle \rightarrow \frac{1}{2}(| 20 \rangle +\sqrt{2}| 11 \rangle +| 02 \rangle ), \quad | 20 \rangle \rightarrow \frac{1}{2}(| 20 \rangle -\sqrt{2}| 11 \rangle +| 02 \rangle ). \end{aligned}$$

So, for the input state $| \Psi _{abq}(t) \rangle$, given in Eq. (20), the output state of the balanced linear coupler can be represented as follows:

33 $$\begin{aligned} | \Psi _{cdq}(t) \rangle&= C_{00g}| 00g \rangle +e^{-i\omega _d t}\left( C_{00e}| 00e \rangle +C^\prime _{10g}| 10g \rangle +C^\prime _{01g}| 01g \rangle \right) \nonumber \\&\quad +e^{-2 i \omega _d t}\left( C^\prime _{10e}| 10e \rangle +C^\prime _{01e}| 01e \rangle +C^\prime _{11g}| 11g \rangle +C^\prime _{20g}| 20g \rangle +C^\prime _{02g}| 02g \rangle \right) , \nonumber \\ \end{aligned}$$

where the superposition coefficients are:

34 $$\begin{aligned} C^\prime _{10g}&= \frac{1}{\sqrt{2}}(C_{10g}+C_{01g}),\nonumber \\ C^\prime _{01g}&= \frac{1}{\sqrt{2}}(C_{10g}-C_{01g}),\nonumber \\ C^\prime _{10e}&= \frac{1}{\sqrt{2}}(C_{10e}+C_{01e}),\nonumber \\ C^\prime _{01e}&= \frac{1}{\sqrt{2}}(C_{10e}-C_{01e}),\nonumber \\ C^\prime _{11g}&= \frac{1}{\sqrt{2}}(C_{20g}-C_{02g}),\nonumber \\ C^\prime _{20g}&= \frac{1}{2}(C_{20g}+\sqrt{2}C_{11g}+C_{02g}),\nonumber \\ C^\prime _{02g}&= \frac{1}{2}(C_{20g}-\sqrt{2}C_{11g}+C_{02g}). \end{aligned}$$

We can calculate the coefficients $C_{n_a,n_b,g/e}$ iteratively^45^. For a single excitation and assuming the resonance case $\Delta _{_\mathrm{SMR}}=\Delta _{m}=\Delta _{q}=\Delta$ and $\kappa _{a}=\kappa _b=\kappa$, the steady-state superposition coefficients can be calculated from:

35 $$\begin{aligned} 0= & {} \left( \Delta -\frac{i\kappa }{2}\right) C_{01g}+f C_{10g}+\eta C_{00g},\nonumber \\ 0= & {} \left( \Delta -\frac{i\kappa }{2}\right) C_{10g}+f C_{01g}+g C_{00e}, \nonumber \\ 0= & {} \left( \Delta -\frac{i\gamma }{2}\right) C_{00e}+g C_{10g}, \end{aligned}$$

where $\eta =\eta _b$, $\Delta =\omega _i-\omega _p$ and $\omega _{_\mathrm{SMR}}=\omega _m=\omega _q=\omega$. Moreover, we assume the weak-driving regime. So, in the first iteration, the contributions from the states with more than a single excitation, such as $C_{01e}$, $C_{11g}$, ..., are negligible. From Eq. (35), by comparing the coefficients with a single excitation, we can see that $C_{10g}$ and $C_{00e}$ are much larger than $C_{01g}$, because of a weak-pump amplitude $\eta$, and they can be written as

36 $$\begin{aligned} C_{10g}=\frac{f(24\Delta -2i\kappa )C_{01g}}{(24g^2-24\Delta ^2+14i\kappa \Delta +\kappa ^2)}\, ,\nonumber \\ C_{00e}=-\frac{24f g C_{01g}}{(24g^2-24\Delta ^2+14i\kappa \Delta +\kappa ^2)}. \end{aligned}$$

In the second iteration, to include states with two excitations in total, the steady-state coefficients can be calculated from:

37 $$\begin{aligned} 0&= 2\Delta _{\kappa } C_{11g}+\sqrt{2}f C_{20g}+\sqrt{2}f C_{02g}+g C_{01e}+\eta C_{10g}, \nonumber \\ 0&= \Delta _{\kappa } C_{10e}+\Delta _{\gamma } C_{10e}+f C_{01e}+\sqrt{2}g C_{20g}, \nonumber \\ 0&= \Delta _{\kappa } C_{01e}+\Delta _{\gamma } C_{01e}+f C_{10e}+g C_{11g}+\eta C_{00e}, \nonumber \\ 0&= 2\Delta _{\kappa } C_{20g}+\sqrt{2}f C_{11g}+\sqrt{2}g C_{10e}, \nonumber \\ 0&= 2\Delta _{\kappa } C_{02g}+\sqrt{2}f C_{11g}+\sqrt{2}\eta C_{01g}, \end{aligned}$$

where $\Delta _{\kappa }=\Delta -i\kappa /2$ and $\Delta _{\gamma }=\Delta -i\gamma /2$. As can be seen from Eq. (37), we have

38 $$\begin{aligned} C_{02g}= -(\sqrt{2}f C_{11g}+\sqrt{2}\eta C_{01g})/(2\Delta _{\kappa }). \end{aligned}$$

So, to minimise $C_{02g}$, the minimalization of $C_{11g}$ and $C_{01g}$ is also required. Destructive interference between the direct and indirect excitation paths in the energy ladders of the total system can enable us minimising $C_{02g}$. This explains the occurrence of the dip in $g_b^{(2)}(0)$ in the mode *b*, as a signature of PB. As clearly seen in Fig. 12a, the optimal PB in this mode occurs at $\Delta _{_\mathrm{SMR}}/g=\pm 1.2$. The above equations lead us to analytical optimal conditions for the system parameters to maximise the sub-Poissonian character of the QD mode and, thus, to optimise the parameters for observing PB in the mode *b*. Given Eq. (39) for a single excitation and Eq. (41) for two excitations, which are calculated from Eq. (37), we show that the second-order correlation function calculated by this method and the master equation method both give very similar predictions, as shown in Fig. 12, where the thick curves are calculated based on the non-Hermitian Hamiltonian approach and the thin curves correspond to the master-equation approach for the modes *a*, *b*, and *c*.

Thus we find

39 $$\begin{aligned} C_{01g}&= (\Delta _{\kappa } \Delta _{\gamma }-g^2)\eta X^{-1}_{5},\nonumber \\ C_{10g}&= -\Delta _{\gamma }f\eta X_{5}^{-1}, \end{aligned}$$

which yields

40 $$\begin{aligned} C^\prime _{10g}= \frac{(\Delta _{\kappa } \Delta _{\gamma }-\Delta _{\gamma }f-g^2)\eta }{\sqrt{2}X_5 }, \end{aligned}$$

Analogously, we find

41 $$\begin{aligned} C_{02g}&= \frac{\eta ^2[-2\Delta _{\kappa }^3\Delta _{\gamma }X_1+\Delta _{\kappa }^2 X_2g^2-X_6 g^4+g^6]}{\sqrt{2}X_5 (X_3-X_4)},\nonumber \\ C_{20g}&= -\frac{\eta ^2 f^2[2\Delta _{\kappa } \Delta _{\gamma }X_1+(2\Delta _{\kappa } - \Delta _{\gamma })\Delta _{\kappa \gamma }g^2-g^4]}{\sqrt{2}X_5 (X_3-X_4)},\nonumber \\ C_{11g}&= \frac{\eta ^2 f(2\Delta _{\kappa }^2 \Delta _{\gamma }X_1+X_7 g^2+\Delta _{\gamma }g^4)}{X_5 (X_3-X_4)}, \end{aligned}$$

where $\Delta _{\kappa \gamma }=\Delta _{\kappa } + \Delta _{\gamma }$ and the auxiliary functions $X_n$ read: $X_1=\Delta _{\kappa \gamma }^2-f^2$, $X_2=\Delta _{\kappa \gamma }(2\Delta _{\kappa } +5\Delta _{\gamma })-4f^2$, $X_3=2\Delta _{\kappa }(\Delta _{\kappa }^2- f^2)X_1$, $X_4=\left[ 3\Delta _{\kappa }^2\Delta _{\kappa \gamma }+(\Delta _{\kappa }-\Delta _{\gamma })f^2\right] g^2-\Delta _{\kappa } g^4$, $X_5=\Delta _{\kappa }^2\Delta _{\gamma }- \Delta _{\gamma }f^2-\Delta _{\kappa } g^2$, $X_6=3\Delta _{\kappa }^2+4\Delta _{\kappa } \Delta _{\gamma }+f^2$, and $X_7=\Delta _{\kappa }(2f^2-3\Delta _{\gamma }\Delta _{\kappa \gamma })$. These formulas, together with $C'_{20g}$ in Eq. (34), enable us to calculate analytically the correlation functions in Eqs. (22) and (23).

## Data Availability

All the data necessary to reproduce the results are included in this published article.

## References

[1] Imamoğlu A, Schmidt H, Woods G, Deutsch M (1997). Strongly interacting photons in a nonlinear cavity. Phys. Rev. Lett..

[2] Miranowicz A, Leoński W, Imoto N (2001). Quantum-optical states in finite-dimensional Hilbert space. I. General formalism. Adv. Chem. Phys..

[3] Leoński W, Miranowicz A (2001). Quantum-optical states in finite-dimensional Hilbert space. II. state generation. Adv. Chem. Phys..

[4] Leoński W, Kowalewska-Kudłaszyk A (2011). Quantum scissors: Finite-dimensional states engineering. Prog. Opt..

[5] Birnbaum KM (2005). Photon blockade in an optical cavity with one trapped atom. Nature (London).

[6] Faraon A (2008). Coherent generation of non-classical light on a chip via photon-induced tunnelling and blockade. Nat. Phys..

[7] Lang C (2011). Observation of resonant photon blockade at microwave frequencies using correlation function measurements. Phys. Rev. Lett..

[8] Hoffman AJ (2011). Dispersive photon blockade in a superconducting circuit. Phys. Rev. Lett..

[9] Reinhard A (2011). Strongly correlated photons on a chip. Nat. Photon..

[10] Müller K (2015). Coherent generation of nonclassical light on chip via detuned photon blockade. Phys. Rev. Lett..

[11] Hamsen C, Tolazzi KN, Wilk T, Rempe G (2017). Two-photon blockade in an atom-driven cavity QED system. Phys. Rev. Lett..

[12] Snijders H (2018). Observation of the unconventional photon blockade. Phys. Rev. Lett..

[13] Vaneph C (2018). Observation of the unconventional photon blockade in the microwave domain. Phys. Rev. Lett..

[14] Majumdar A, Bajcsy M, Vučković J (2012). Probing the ladder of dressed states and nonclassical light generation in quantum-dot–cavity QED. Phys. Rev. A.

[15] Peyronel T (2012). Quantum nonlinear optics with single photons enabled by strongly interacting atoms. Nature (London).

[16] Dayan B (2008). A photon turnstile dynamically regulated by one atom. Science.

[17] Tian L, Carmichael HJ (1992). Quantum trajectory simulations of two-state behavior in an optical cavity containing one atom. Phys. Rev. A.

[18] Leoński W, Tanaś R (1994). Possibility of producing the one-photon state in a kicked cavity with a nonlinear Kerr medium. Phys. Rev. A.

[19] Miranowicz A, Leoński W, Dyrting S, Tanaś R (1996). Quantum state engineering in finite-dimensional Hilbert space. Acta Phys. Slov..

[20] Paul H (1982). Photon antibunching. Rev. Mod. Phys..

[21] Teich MC, Saleh BEA (1988). Photon bunching and antibunching. Prog. Opt..

[22] Kozierowski M (1980). Photon antibunching in nonlinear optical phenomena. Kvantovaya Elektron..

[23] Michler P (2000). A quantum dot single-photon turnstile device. Science.

[24] Wang X, Miranowicz A, Li H-R, Nori F (2016). Multiple-output microwave single-photon source using superconducting circuits with longitudinal and transverse couplings. Phys. Rev. A.

[25] Shamailov S, Parkins A, Collett M, Carmichael H (2010). Multi-photon blockade and dressing of the dressed states. Opt. Commun..

[26] Miranowicz A, Paprzycka M, Liu Y-X, Bajer J, Nori F (2013). Two-photon and three-photon blockades in driven nonlinear systems. Phys. Rev. A.

[27] Chakram, S. et al. Multimode photon blockade. arXiv preprint (2020). arXiv:2010.15292.

[28] Liew TCH, Savona V (2010). Single photons from coupled quantum modes. Phys. Rev. Lett..

[29] Huang R, Miranowicz A, Liao J-Q, Nori F, Jing H (2018). Nonreciprocal photon blockade. Phys. Rev. Lett..

[30] Li B, Huang R, Xu X, Miranowicz A, Jing H (2019). Nonreciprocal unconventional photon blockade in a spinning optomechanical system. Photon. Res..

[31] Yang P (2019). Realization of Nonlinear Optical Nonreciprocity on a Few-Photon Level Based on Atoms Strongly Coupled to an Asymmetric Cavity. Phys. Rev. Lett..

[32] Miranowicz A (2014). State-dependent photon blockade via quantum-reservoir engineering. Phys. Rev. A.

[33] Huang, R. *et al.* Exceptional photon blockade: Engineering photon blockade with chiral exceptional points. *Laser Photonics Rev.* **16**, 2100430 (2022).

[34] Pegg DT, Phillips LS, Barnett SM (1998). Optical State Truncation by Projection Synthesis. Phys. Rev. Lett..

[35] Özdemir SK, Miranowicz A, Koashi M, Imoto N (2001). Quantum-scissors device for optical state truncation: A proposal for practical realization. Phys. Rev. A.

[36] Özdemir SK, Miranowicz A, Koashi M, Imoto N (2002). Pulse-mode quantum projection synthesis: Effects of mode mismatch on optical state truncation and preparation. Phys. Rev. A.

[37] Babichev SA, Ries J, Lvovsky AI (2003). Quantum scissors: Teleportation of single-mode optical states by means of a nonlocal single photon. EPL (Europhys. Lett.).

[38] Koniorczyk M, Kurucz Z, Gábris A, Janszky J (2000). General optical state truncation and its teleportation. Phys. Rev. A.

[39] Miranowicz A (2005). Optical-state truncation and teleportation of qudits by conditional eight-port interferometry. J. Opt. B: Quant. Semicl. Opt..

[40] Miranowicz A, Paprzycka M, Pathak A, Nori F (2014). Phase-space interference of states optically truncated by quantum scissors. Phys. Rev. A.

[41] Reck M, Zeilinger A, Bernstein HJ, Bertani P (1994). Experimental realization of any discrete unitary operator. Phys. Rev. Lett..

[42] Miranowicz A, Özdemir SK, Bajer J, Koashi M, Imoto N (2007). Selective truncations of an optical state using projection synthesis. J. Opt. Soc. Am. B.

[43] Leoński W, Miranowicz A (2004). Kerr nonlinear coupler and entanglement. J. Opt. B.

[44] Miranowicz A, Leoński W (2006). Two-mode optical state truncation and generation of maximally entangled states in pumped nonlinear couplers. J. Phys. B.

[45] Bamba M, Imamoğlu A, Carusotto I, Ciuti C (2011). Origin of strong photon antibunching in weakly nonlinear photonic molecules. Phys. Rev. A.

[46] Flayac H, Savona V (2017). Unconventional photon blockade. Phys. Rev. A.

[47] Liu Y-X (2010). Qubit-induced phonon blockade as a signature of quantum behavior in nanomechanical resonators. Phys. Rev. A.

[48] Didier N, Pugnetti S, Blanter YM, Fazio R (2011). Detecting phonon blockade with photons. Phys. Rev. B.

[49] Wang X, Miranowicz A, Li H-R, Nori F (2016). Method for observing robust and tunable phonon blockade in a nanomechanical resonator coupled to a charge qubit. Phys. Rev. A.

[50] Miranowicz A, Bajer J, Lambert N, Liu Y-X, Nori F (2016). Tunable multiphonon blockade in coupled nanomechanical resonators. Phys. Rev. A.

[51] Shi H-Q, Zhou X-T, Xu X-W, Liu N-H (2018). Tunable phonon blockade in quadratically coupled optomechanical systems. Sci. Rep..

[52] Liu YX, Xu XW, Miranowicz A, Nori F (2014). From blockade to transparency: Controllable photon transmission through a circuit-QED system. Phys. Rev. A.

[53] Kowalewska-Kudłaszyk A, Abo SI, Chimczak G, Peřina J, Nori F, Miranowicz A (2019). Two-photon blockade and photon-induced tunneling generated by squeezing. Phys. Rev. A.

[54] Aspelmeyer M, Kippenberg TJ, Marquardt F (2014). Cavity optomechanics. Rev. Mod. Phys..

[55] Xu X-W, Shi H-Q, Liao J-Q, Chen A-X (2019). Generation of single entangled photon-phonon pairs via an atom-photon-phonon interaction. Phys. Rev. A.

[56] Xu X-W, Shi H-Q, Chen A-X, Liu xY (2018). Cross-correlation between photons and phonons in quadratically coupled optomechanical systems. Phys. Rev. A.

[57] Zhai C, Huang R, Jing H, Kuang L-M (2019). Mechanical switch of photon blockade and photon-induced tunneling. Opt. Express.

[58] Santori C, Pelton M, Solomon G, Dale Y, Yamamoto Y (2001). Triggered single photons from a quantum dot. Phys. Rev. Lett..

[59] Ding X (2016). On-demand single photons with high extraction efficiency and near-unity indistinguishability from a resonantly driven quantum dot in a micropillar. Phys. Rev. Lett..

[60] Grangier P, Walls DF, Gheri KM (1998). Comment on strongly interacting photons in a nonlinear cavity. Phys. Rev. Lett..

[61] Kimble HJ (1998). Strong interactions of single atoms and photons in cavity QED. Phys. Scripta.

[62] Gu X, Kockum AF, Miranowicz A, Liu Y-X, Nori F (2017). Microwave photonics with superconducting quantum circuits. Phys. Rep..

[63] Tian L (2009). Ground state cooling of a nanomechanical resonator via parametric linear coupling. Phys. Rev. B.

[64] Kockum AF, Miranowicz A, Liberato SD, Savasta S, Nori F (2019). Ultrastrong coupling between light and matter. Nat. Rev. Phys..

[65] Restrepo J, Ciuti C, Favero I (2014). Single-polariton optomechanics. Phys. Rev. Lett..

[66] Larson J, Mavrogordatos T (2021). The Jaynes–Cummings Model and Its Descendants.

[67] Ridolfo A, Leib M, Savasta S, Hartmann MJ (2012). Photon blockade in the ultrastrong coupling regime. Phys. Rev. Lett..

[68] Garziano L (2015). Multiphoton quantum rabi oscillations in ultrastrong cavity qed. Phys. Rev. A.

[69] Mercurio, A., Abo, S., Mauceri, F., Russo, E., Macri, V., Miranowicz, A., Savasta, S. & Di Stefano, O. Pure dephasing of light-matter systems in the ultrastrong and deep-strong coupling regimes (2022). arXiv:2205.05352. 10.1103/PhysRevLett.130.123601 37027872

[70] Sánchez Muñoz C, Frisk Kockum A, Miranowicz A, Nori F (2020). Simulating ultrastrong-coupling processes breaking parity conservation in Jaynes-Cummings systems. Phys. Rev. A.

[71] Kuhn A (2015). Cavity Induced Interfacing of Atoms and Light.

[72] Mandel L, Wolf E (1995). Optical Coherence and Quantum Optics.

[73] Kimble HJ, Dagenais M, Mandel L (1977). Photon antibunching in resonance fluorescence. Phys. Rev. Lett..

[74] Verhagen E, Deléglise S, Weis S, Schliesser A, Kippenberg TJ (2012). Quantum-coherent coupling of a mechanical oscillator to an optical cavity mode. Nature (London).

[75] Walls DF, Milburn GJ (1994). Quantum Optics.

[76] Kubanek A (2008). Two-photon gateway in one-atom cavity quantum electrodynamics. Phys. Rev. Lett..

[77] Minganti F, Miranowicz A, Chhajlany RW, Nori F (2019). Quantum exceptional points of non-Hermitian Hamiltonians and Liouvillians: The effects of quantum jumps. Phys. Rev. A.

[78] Minganti F, Miranowicz A, Chhajlany RW, Arkhipov II, Nori F (2020). Hybrid-Liouvillian formalism connecting exceptional points of non-Hermitian Hamiltonians and Liouvillians via postselection of quantum trajectories. Phys. Rev. A.

[79] Zou XT, Mandel L (1990). Photon-antibunching and sub-Poissonian photon statistics. Phys. Rev. A.

[80] Miranowicz A, Bartkowiak M, Wang X, Liu Y-X, Nori F (2010). Testing nonclassicality in multimode fields: A unified derivation of classical inequalities. Phys. Rev. A.

[81] Hong S (2017). Hanbury Brown and Twiss interferometry of single phonons from an optomechanical resonator. Science.

[82] Shchukin EV, Vogel W (2005). Nonclassical moments and their measurement. Phys. Rev. A.

[83] Shchukin E, Vogel W (2006). Universal measurement of quantum correlations of radiation. Phys. Rev. Lett..

